# Programmable next-generation supramolecular self-assembled materials as drug delivery systems

**DOI:** 10.1016/j.bioactmat.2026.01.040

**Published:** 2026-02-03

**Authors:** Hongwei Fu, Weihao Gao, Yixuan Tang, Xiangli Liu, Mi Wang, Jichuan Zhang, Tianqi Liu, Jiaheng Zhang

**Affiliations:** a School of Chemistry and Chemical Engineering, Nanjing University of Science and Technology, Nanjing, 210094, PR China; b Research Centre of Printed Flexible Electronics, School of Materials Science and Engineering, Harbin Institute of Technology (Shenzhen), Shenzhen, 518055, PR China; c Sauvage Laboratory for Smart Materials, School of Materials Science and Engineering, Harbin Institute of Technology (Shenzhen), Shenzhen, 518055, PR China; d School of Biomedical Engineering, Harbin Institute of Technology (Shenzhen), Shenzhen, 518055, PR China

**Keywords:** Supramolecular, Self-assembled materials, Drug delivery systems, Programmability, Nanomedicines

## Abstract

Supramolecular self-assembly, driven by noncovalent interactions, serves as a fundamental principle for constructing complex functional systems (biological membranes, proteins, and DNA). Inspired by these natural paradigms, biomimetic self-assembly strategies have promoted the development of drug delivery systems (DDSs), including liposomes, lipid nanoparticles (LNPs), and virus-like particles. While these nanomedicines are clinically established, they continue to face persistent challenges, including maintaining drug stability and activity, achieving targeted delivery, overcoming biological barriers, and ensuring efficient intracellular release. Overcoming these obstacles requires the rational design of next-generation drug delivery materials. In this review, a comprehensive analysis of supramolecular self-assembled materials (SAMs) is provided. It traces the development and self-assembly mechanisms of SAMs. It defines the characteristics of next-generation SAMs, centered on their programmability, and focuses on introducing the innovative design strategy guidelines for next-generation SAMs. These guidelines fully embody the high programmability, precise size/morphology control capabilities, and multifunctional properties of SAMs. Furthermore, it discusses the latest application progress of SAMs in treating various diseases, and emphasizes the future strategies and challenges of SAM-based DDSs, aiming to facilitate broader clinical applications and benefit human health.

## Highlights

• First definition of the biological properties of next-generation supramolecular SAMs.
• Supramolecular self-assembly technology as effective strategy for developing next-generation SAMs-based DDS.
• Comprehensive analysis of next-generation SAMs: evolution, applications mechanisms.
• Next-generation SAMs highlights programmability, precise size control, and multifunctional integration.
• Next-generation SAMs for targeted therapy use advanced design to tackle DDS challenges.

## Graphical abstract

This review analyzes innovative drug delivery platforms: supramolecular self-assembled materials (SAMs) for addressing challenges in nanomedicine. This review systematically categorizes next-generation SAMs and discusses design strategies that emphasize their programmability, precise size/morphology control, and multifunctional integration. Finally, summarizing the application of SAM for diseases treatment and proposing future strategies to accelerate clinical translation.**Next-generation supramolecular SAMs programmatically design functional integrated DDS for disease treatment**.

**Figure:**
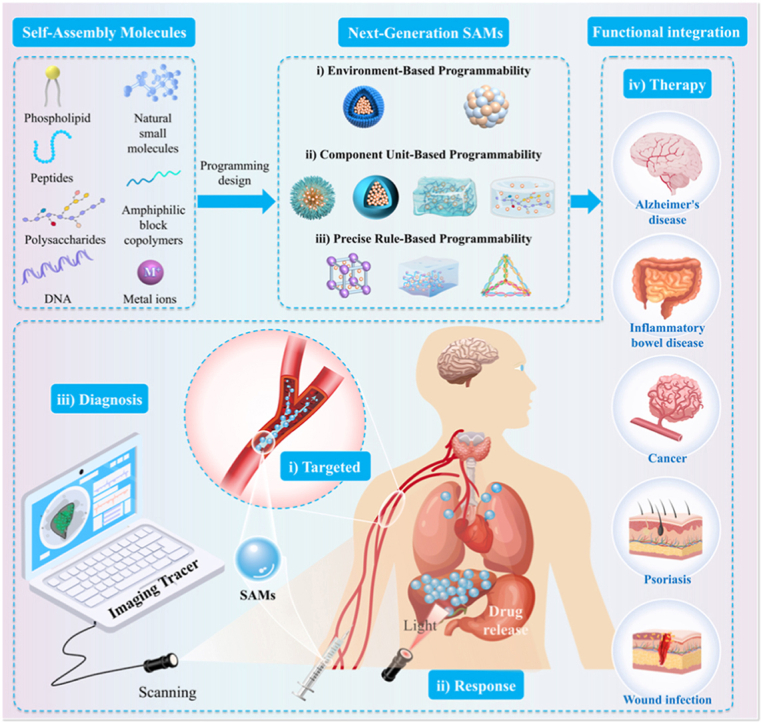
**Next-generation supramolecular SAMs programmatically design functional integrated DDS for disease treatment**.

## 1 Introduction

In 1987, a Nobel Laureate in Chemistry Jean-Marie Lehn proposed: “Covalent bonds underpin the field of molecular chemistry; molecular assemblies and intermolecular bonds underpin the field of supramolecular chemistry.” [1] The core of supramolecular chemistry lies in constructing higher-order structures with specific functions through noncovalent molecular interactions. Crown ethers and cryptands first achieved molecular recognition-driven self-assembly, revealing the high selectivity of host-guest chemistry. The shift from molecular recognition to self-assembly studies marks the transition from observing phenomena to rational design [[2], [3], [4]]. Supramolecular self-assembly is crucial for constructing complex materials and functions as a fundamental rule in natural systems. For example, biological membranes form through phospholipid self-assembly, creating a selectively permeable bilayer barrier; Proteins form higher-order structures with specific bioactivity through the spontaneous folding and assembly of polypeptide chains; DNA stores genetic information through strict base complementary pairing. Poglazov's 1967 study of phage assembly revealed how viruses form complete particles within host cells [5,6]. Inspired by biological self-assembly, scientists have employed nature's assembly principles to create biomimetic systems with complex structures and tailored properties [7]. Liposomes and LNPs, which mimic the phospholipid bilayer structure of biological membranes, have become effective drug delivery carriers [[8], [9], [10]]; Virus-like particles (VLPs), formed through the precise self-assembly of viral capsid proteins, provide non-replicative, highly efficient delivery platforms [11]; DNA nanotechnology, leveraging the highly programmable nature of base pairing, enables atomic-level precise nanostructure construction through base pairing [12].

After over seventy years of development, self-assembly delivery technologies like liposomes, LNPs, VLPs, and pharmaceutical co-crystals have become increasingly mature and demonstrated value in clinical treatment [13]. Despite this progress, these drug delivery technologies continue to face various bottleneck challenges which vary by drug type. The main challenges include: (i) ensuring drug molecule activity and stability; (ii) enhancing targeted distribution to diseased tissues and cells; (iii) promoting drug transport across biological barriers; (iv) optimizing intracellular drug transport and release [[14], [15], [16], [17], [18]]. Drug delivery research aims to develop formulations that deliver drugs to predefined targets with specific release kinetics and duration. Future DDS research will increasingly focus on chronic diseases, with small molecule drugs and biologics reaching equal shares in the market. And, research will emphasize targeted, long-acting formulations that can efficiently cross biological barriers [13]. To overcome these challenges, research on novel SAMs based on DNA, peptides, polysaccharides, metal-organic frameworks (MOFs) and other materials has increased [[19], [20], [21], [22]]. These materials, through their programmable molecular structure, controllable particle properties, and functional integration capabilities, show potential exceeding traditional systems.

However, despite rapid progress in laboratory research, industrialization of these advanced materials has lagged. Novel SAMs combining functionality, targeting ability, and environment responsiveness remain unused in clinical practice. The core challenge remains understanding how to precisely regulate self-assembly/disassembly processes and morphological evolution of the resulting assemblies based on molecular structure [23].

**Fig. 1:**
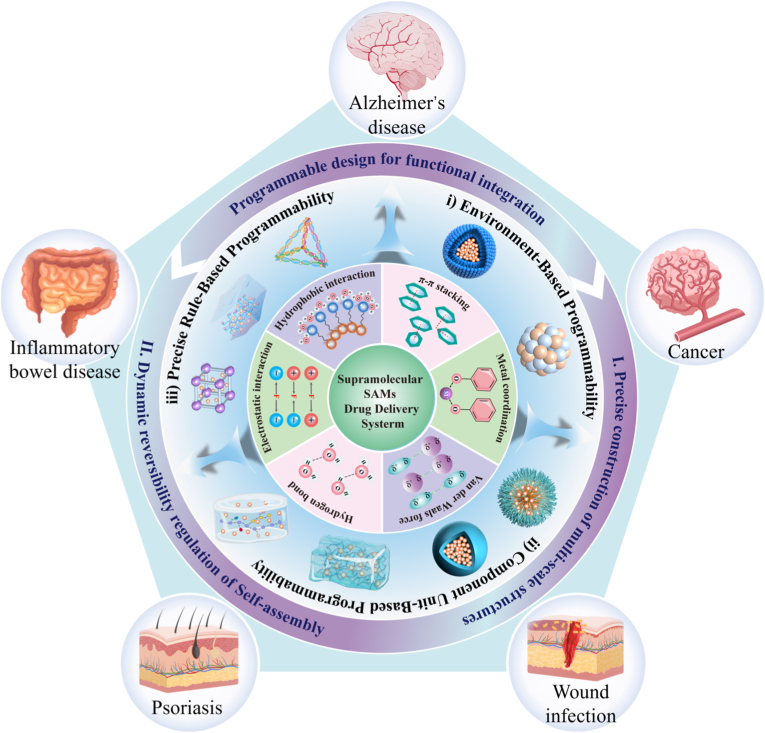
Schematic for the study of supramolecular SAMs-based DDS.

This review focuses on next-generation supramolecular SAMs (Fig. 1) in DDSs, reviewing the development, assembly mechanisms, programming strategies, and therapeutic value. Existing reviews often overlook the common design logic of different material systems. This review first proposes a programmability-centered unified theoretical framework. Based on this, it defines next-generation SAMs' core characteristics: intelligent DDSs evolving from passive adaptation to active molecular programming, from single-signal response to multimodal synergy, and from single-function to system integration. Guided by this framework, SAMs' programmability is classified into three levels: environment-based, component unit-based, and precise rule-based. Reviewing these three levels clarifies that SAMs’ programmability and modular assembly are the core drivers of nanomedicine evolution from passive carriers to intelligent devices. Building on this, the review systematizes material property-application mappings across biological barriers, builds a design-function-application loop, and offers theoretical support and a blueprint for novel DDSs.

## 2 Programmable category of Supramolecular Self-assembled materials

**Fig. 2:**
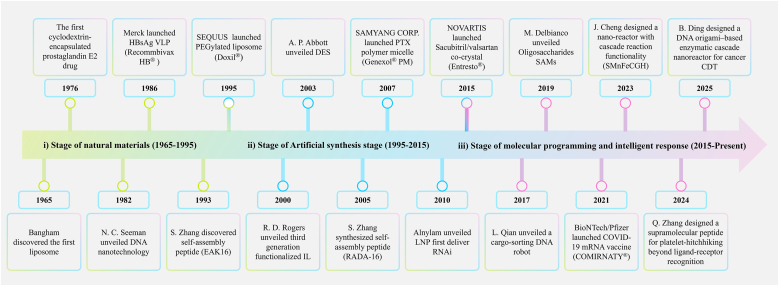
Development timeline of common supramolecular SAMs DDSs (combining the clinical products). 1965 [33], 1976 [34], 1982 [35], 1986 [36], 1993 [37], 1995 [38], 2000 [39], 2003 [40], 2005 [41], 2007 [42], 2010 [43], 2015 [44], 2017 [45], 2019 [46], 2020 [47], 2023 [48], 2024 [49], 2025 [50].

Since the concept of supramolecular chemistry was proposed, the Nobel Prizes in Chemistry in 1987, 2016, and 2025 have propelled the development of supramolecular chemistry to unprecedented heights [[24], [25], [26]]. Supramolecular self-assembly technology is driving the evolution of drug delivery materials, progressing from natural sources to artificial synthesis, and further to molecular programming and intelligent response. This developmental trajectory can be divided into three main stages (Fig. 2):

i. **The Stage of Natural Source (1965**–**1995).** Supramolecular self-assembly phenomena are ubiquitous in nature, providing abundant natural components as building blocks for self-assembled materials (e.g., lipids, peptides, proteins, nucleic acids, and cyclodextrins). At this stage, natural or biomimetic self-assembled structures were primarily used as passive drug carriers (such as liposomes delivering small-molecule drugs, virus-like particles for vaccines) or structural support materials. These materials validated the potential of biological self-assembly in drug delivery systems, but their design was constrained by the inherent properties of natural substances. They lacked artificial regulatory capabilities, which resulted in insufficient precision and functionality.
ii. **The Stage of Artificial Synthesis (1995**–**2015).** This stage is centered on the integration of synthetic chemistry and self-assembly engineering, shifting the focus to rationally designed artificial carriers. On one hand, researchers performed engineering modifications on natural structures (e.g., PEGylated lipids to prolong circulation time; introducing ionizable cationic lipids to prepare LNPs for drug protection and targeted release). On the other hand, novel synthetic designs were developed: for example, Professor Zhang's team, based on analyzing the principles of natural self-assembling peptides, designed a novel peptide RADA16, laying the foundation for peptide-based carrier development; Intermolecular matching strategies were adopted to synthesize diverse assemblies such as block copolymers, bioactive ionic liquids, and pharmaceutical co-crystals. Synthetic materials have driven extensive clinical translation, but their self-assembly mechanisms are mostly pre-set, lacking molecular-level programmability.
iii. **The Stage of Molecular Programming and Intelligent Response (2015–Present).** At present, first-principles serve as the underlying logic for material design, enabling programmable molecular design via theoretical calculations and simulations. Guided by biological regularity principles (e.g., ligand-receptor pairing, base complementary pairing, metal coordination, and stimulus responsiveness), system functions can be precisely tailored starting from molecular structures. Current research focuses on enhancing dynamic adaptability and precise regulation capabilities, aiming to construct intelligent DDSs, thereby laying the groundwork for personalized medicine and scenario-responsive therapies.

SAMs are gradually evolving into novel DDSs, with programmability being the core to upgrading supramolecular drug delivery systems into multifunctional intelligent platforms. The following presents a hierarchical classification of their programmability:

### 2.1 Environment-based programmability

This is the primary form, applicable to naturally derived or simply synthesized molecules (e.g., lipids, bioactive small molecules, sugars). The inherent structure of the material is fixed, and by adjusting external thermodynamic parameters (e.g., pH, ionic strength, solvent polarity) to alter the strength of intermolecular forces, the switching of self-assembled morphology and function is achieved. Examples include the formation of phospholipid bilayers in aqueous solutions, conformational changes of proteins at their isoelectric points, and the regulation of the 3D structures of polysaccharides, nucleic acids, and proteins—all of which are dependent on environmental parameters. High protein concentrations promote β-sheet formation in spider silk inspired protein, as seen in experiments where evaporation induces interfacial crystallization [27]. Moreover, the IL (1-ethyl-3-methylimidazolium chloride, [BMIM][Cl]) breaks silk fibroin's intramolecular hydrogen bonds through solvent effect, promoting protein chain unfolding and reorganizing them into porous microspheres [28].

### 2.2 Component unit-based programmability

This level actively encodes design freedom through monomer sequence engineering or component ratio adjustment, with typical representatives including polypeptides, block copolymers, and multi-component assemblies. Polypeptides preset self-assembly tendencies via alternating "hydrophobic-hydrophilic" residue sequences [29]; block copolymers regulate phase separation through A/B block length ratios [30]; multi-component systems (e.g., ionic liquids) achieve directed assembly *via* ion-complementary recognition sites [31]. Through molecular design and synthesis, assembly logic is preset at the molecular scale for this level, greatly expanding the sources of materials and laying a solid foundation for their development.

### 2.3 Precise rule-based programmability

This level enables intelligent programming *via* precise molecular pairing rules (e.g., A-T/C-G base complementarity, metal coordination), endowing materials with multifunctionality and facilitating the construction of intelligent drug delivery systems. Metal coordination drives metal nodes and organic ligands to form high-dimensional ordered frameworks (e.g., MOFs), whose high specific surface area and designable pores exhibit significant application potential. DNA nanotechnology is based on base complementarity to design 2D/3D nanostructures [32].

In summary, the core value of this hierarchical framework lies in revealing the characteristics of next-generation self-assembled materials through the progressive hierarchy, proceeding from environmental parameters *via* component encoding to precise molecular pairing rules. Specifically, it defines a fundamental modal transformation in supramolecular self-assembled materials: from passive environmental adaptation to active molecular programming, from single-signal responsiveness to multimodal synergy, and from monofunctionality to system integration. This progression thereby drives drug delivery to evolve from a drug-loading vehicle into an intelligent system.

## 3 Driving forces of Supramolecular Self-assembly

**Fig. 3:**
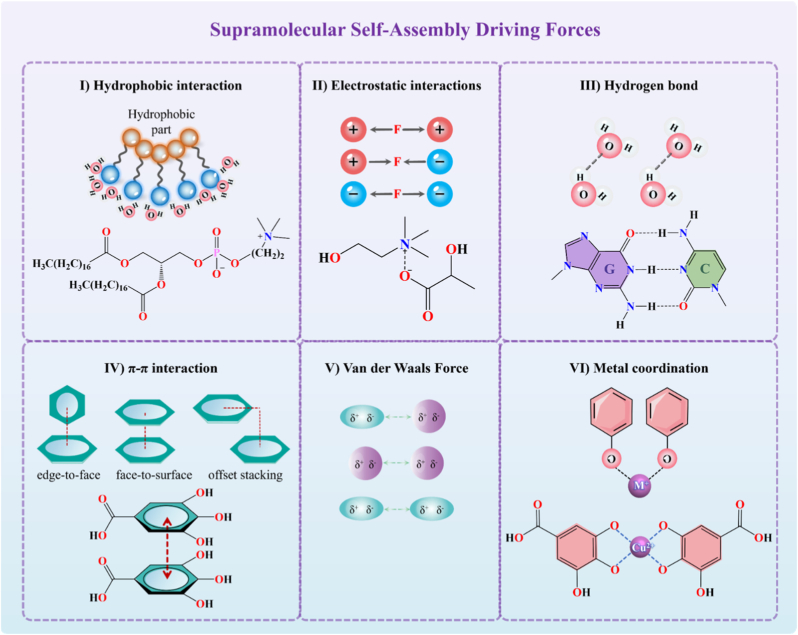
Types of major supramolecular interactions that drive the self-assembly process, including hydrophobic interaction, electrostatic interaction, hydrogen bond, van der Waals force, metal coordination, and *π*–*π* stacking.

Self-assembly is a prevalent phenomenon in nature. This section provides a concise overview of the primary driving forces behind supramolecular SAMs (Fig. 3). It briefly elucidates the roles that each driving force plays in supramolecular self-assembly. The core driving force of supramolecular self-assembly originates from the synergistic effect of multiple weak interaction forces. These interaction forces, driving self-assembly, have lower bond energies (0–400 kJ/mol), allowing them to be disrupted and reformed with a small energy input, exhibiting reversibility and dynamic responsiveness [51,52]. These characteristics are crucial in the self-assembly process. The main driving forces in supramolecular self-assembly are as follows (see Table 1 for a comparative analysis):

**Table 1:** Comparative analysis of primary driving forces in supramolecular self-assembly.

Driving Force	Bond Energy (kJ·mol^−1^)	Characteristics	Representative Functional Groups	Primary Role in SAMs
Hydrophobic Interaction	difficult to assess	Solvent-dependent, Entropy-driven	Long alkyl chains, Aromatic rings (Phe, Trp residues)	Aggregation Initiator: Acts as the primary thermodynamic force driving amphiphiles to form micelles/vesicles in aqueous media
Electrostatic Interaction	250	Non-directional, Medium selectivity	Ammonium (NH_3_^+^), Phosphate (PO_4_^−^), Carboxyl (COO^−^)	Ion-based assembly driver: Forming polyelectrolyte complexes and ionic liquid assemblies
Hydrogen Bond	10–65	Directionality, Specificity, Reversibility,	Amide (-CONH-), Hydroxyl (-OH), DNA Bases (A-T/G-C)	Directed assembly: Forming hydrogen bond networks to construct two or three-dimensional assembly structures
π-π Stacking	0–50	High selectivity, High spatiality	Aromatic rings (Benzene, Indole), Conjugated systems	Core Stabilization: Stabilizes the loading of planar aromatic drugs and reinforces peptide nanofiber backbones
Van der Waals Force	<5	Proximity-dependent, Between any molecules	All atoms (fluctuating dipoles)	Auxiliary Packing: maintaining the structural stability of supramolecular assemblies
Metal-Ligand Coordination	0–400	High directionality and selectivity, High intensity	Carboxyl (-COOH), Imidazole, Phenolic hydroxyl (-OH)	Structural Rigidification: Dictates the long-range order and permanent porosity of crystalline frameworks

**Ⅰ) Hydrophobic interaction:** Non-polar molecules interact in polar environments to minimize contact with water by forming aggregates. This interaction spontaneously drives the formation of ordered structures like micelles and vesicles by amphiphilic molecules. Phospholipid molecules with one hydrophilic end and one hydrophobic end can self-assemble into micelles and vesicles through hydrophobic interaction [53]. SAMs have broad applications in drug delivery, and researchers are designing peptides [54] and polymer molecules [55] with special amphiphilic structures based on this principle.

**Ⅱ) Electrostatic interaction:** This interaction occurs between charged molecules or groups, showing repulsion between like charges and attraction between opposite charges. It enables self-assembly of ion-complementary peptides with positively charged groups (lysine and arginine) and negatively charged groups (aspartic acid and glutamic acid). Specific cations and anions can form liquid salts at room temperature, which have extensive applications in transdermal drug delivery [56].

**Ⅲ) Hydrogen bond:** Hydrogen bonds form between a hydrogen atom and two electronegative atoms, typically in the A-H···B system. As a directional and saturable self-assembly driving force, hydrogen bonds enhance the structural stability of supramolecular structures and guide the direction of molecular self-assembly. For example, β-sheet secondary structures in proteins are stabilized through parallel or antiparallel interchain hydrogen bond arrays. While DNA nanostructures is maintained through hydrogen bond-driven base complementary pairing [32]. In DES, choline chloride and urea form three-dimensional structures through N-H···Cl and O-H···O hydrogen bond networks [57].

**Ⅳ) π-π stacking:** π-π stacking is the interaction between aromatic rings with π-orbitals, classified into edge-to-face stacked, offset, and face-to-surface stacked modes [58]. The π-electron clouds of aromatic molecules attract each other to form stable structures. This interaction is widely present in the self-assembly of biomolecules containing π-systems [59].

**Ⅴ) Van der Waals force:** Van der Waals forces are the weakest non-covalent interactions (0.5–3.0 kJ/mol) with three components: orientation (Keesom), induction (Debye), and dispersion (London) [60]. Although individual van der Waals forces are weak, their cumulative effect significantly enhances the stability of supramolecular structures when molecules aggregate on a large scale.

**Ⅵ) Metal-ligand coordination:** Metal coordination exists between metal ions and organic ligands, with metals serving as central cross-linking points to form complex supramolecular structures. Polyphenolic compounds can coordinate with metals to form metal-polyphenol networks [61]. Furthermore, typical MOFs can be used for drug loading and release [62].

## 4 Programmability of next-generation Supramolecular Self-assembled drug delivery materials

### 4.1 Environment-based programmability

#### 4.1.1 Lipid nanoparticles

**Fig. 4:**
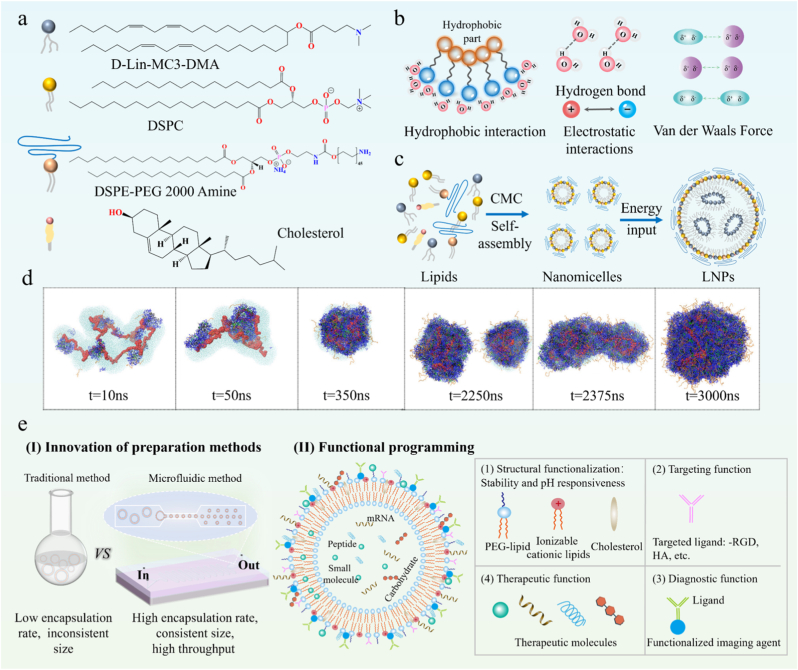
LNPs-based SAM as DDS. **a)** Structural diagram of LNPs components **b)** Driving force for self-assembly of phospholipid molecules. **c)** The process of phospholipid molecule self-assembly undergoes a morphological transformation. **d)** Molecular dynamics simulation of the self-assembly process of LNPs. Adapted with permission [68]. Copyright 2024, American Chemical Society. **e)** Design and optimization strategies for lipid SAMs drug delivery systems.

LNPs have emerged as a next-generation DDS to address the critical clinical challenge of delivering nucleic acids (e.g., mRNA, siRNA), which face degradation in circulation, poor cellular uptake, and inefficient endosomal escape. To tackle these issues, LNPs employ engineered multicomponent core–shell nanostructures comprising ionizable cationic lipids, helper lipids, and steric-stabilizing PEG-lipid conjugates (Fig. 4a). Phospholipids, the main components of LNPs, have hydrophilic heads and hydrophobic tails linked by ester bonds. Structurally, LNPs possess a self-assembled membrane structure, formed by lipid molecules analogous to those found in biological membranes. These structures include micelles, bilayers, and vesicles, primarily driven by hydrophobic interaction and with additional contributions from non-covalent bonds such as hydrogen bonds, electrostatic interactions, and van der Waals force (Fig. 4b). Phosphatidylcholines adopt bilayer configurations in aqueous environments, with hydrophobic acyl chains sequestered inward and hydrophilic headgroups exposed to water. The enthalpy-entropy compensation plays a critical role in the self-assembly process of dipalmitoyl-phosphatidylcholine (DPPC), where water interactions and Coulomb interactions between choline and lipid phosphate fragments promote the process through the enthalpy term, while the restricted mobility of lipids within the bilayer impedes the process through the entropy term [8]. Helper lipids cholesterol and distearoyl phosphatidylcholine (DSPC) form lipid raft domains through van der Waals forces and hydrophobic interaction, enhancing the rigidity and stability of the bicontinuous lipid bilayer. This design directly improves mRNA payload and protection against degradation in circulation [63]. The lipid bilayer's flexibility enables morphological transitions. At critical micellar concentrations (CMCs), lipids aggregate to micelles, which can become planar bilayers upon solvent removal or energy input, and eventually enclose vesicles (Fig. 4c) [64,65]. For example, microfluidic-mediated oil phase and water phase mixing, combined with ethanol diffusion, and triggered phospholipid reorganization from disordered micelles to unilamellar vesicles [66]. This phenomenon has been verified in molecular simulations (Fig. 4d).

LNPs exhibit colloidal stability and payload protection, achieving nucleic acid encapsulation efficiencies above 90 % while enabling spatiotemporal control over bioactive cargo release [67]. Computational modeling reveals that nano structural polymorphism is not random but a programmed feature: at low pH (acidic endosomes), LNPs adopt an inverted micelle structure (design choice for pH responsiveness), maximizing mRNA encapsulation and stability; upon reaching neutral pH (cytoplasm), they transition to a “bleb” structure, which reduces membrane curvature and enhances mRNA retention for efficient translation. Notably, mRNA-encapsulated LNPs constructed from pH-responsive ionizable lipids DLin-MC3-DMA transition from inverted micelle (low pH) to “bleb” structure (neutral pH). Protonation-mediated endosomal membrane disruption facilitates cytoplasmic payload delivery [67,68]. Lipid phase segregation modulates intracellular trafficking pathways, suggesting design rules for optimizing endosomal escape. To promote the innovative development of LNP, optimization programming strategies for SAMs design are summarized as follows (Fig. 4e):

I) Innovation of preparation methods: Traditional self-assembly preparation had simple operation but low efficiency and inconsistent particle size. Innovations in preparation methods overcome these limitations, enhancing precision and leading to significant advancements in DDS. Manufacturing innovations, such as microfluidics technology, enable high-throughput production of monodisperse nanoparticles with tunable biodistribution profiles [69]. Smaller particles extravasate into tumors more efficiently, directly enhancing therapeutic efficacy in solid tumor models. DNA nano templating guides lipid self-assembly into monodisperse LNPs with programmable sizes, highlighting the role of external confinement in overcoming kinetic barriers during vesicle formation [65].
II) Functionalization programming: Programmed design endows materials with new functions for multifunctional assemblies. Stimulus-responsive ligands (e.g., pH-cleavable PEG) or targeting moieties (e.g., RGD) can be used for spatiotemporal control. Environment-responsive release occurs through pH-sensitive components [67], enzymatic degradation [70], or thermotropic phase transitions [71]. Response processes combine thermodynamic and kinetic alterations, such as lipid packing density and membrane fluidity, further modulating drug retention and release profiles [72]. Advanced targeting uses chimeric nanobody-conjugated formulations exhibiting HER2-specific tropism through surface-immobilized antigen-binding domains, demonstrating ligand-directed cellular internalization via receptor-mediated endocytosis [53]. Additionally, the construction of composite systems enhances single systems functions and biocompatibility. Combining lipid and polypeptide vectors improves transfection efficiency of single vectors. In composite vectors, peptides facilitate nucleic acid compression and endosomal escape, while lipids enhance cellular uptake and stability. By optimizing peptide design, lysine (K) compresses nucleic acids through electrostatic interaction. Histidine (H) protonation in acidic endosomes induces “proton sponge effect”, and ruptures endosomal membrane. The hydrophobic ends of leucine (L) and phenylalanine (F) promote self-assembly with lipids and enhance cell membrane interactions [71].

In summary, LNPs exemplify how rational design choices including ionizable lipid chemistry, morphology programming and functionalization translate into specific functions, which ultimately address the clinical challenges of nucleic acid drug delivery and enable breakthroughs in mRNA vaccines, gene editing and personalized medicine.

#### 4.1.2 Natural carrier-free assembled materials

**Fig. 5:**
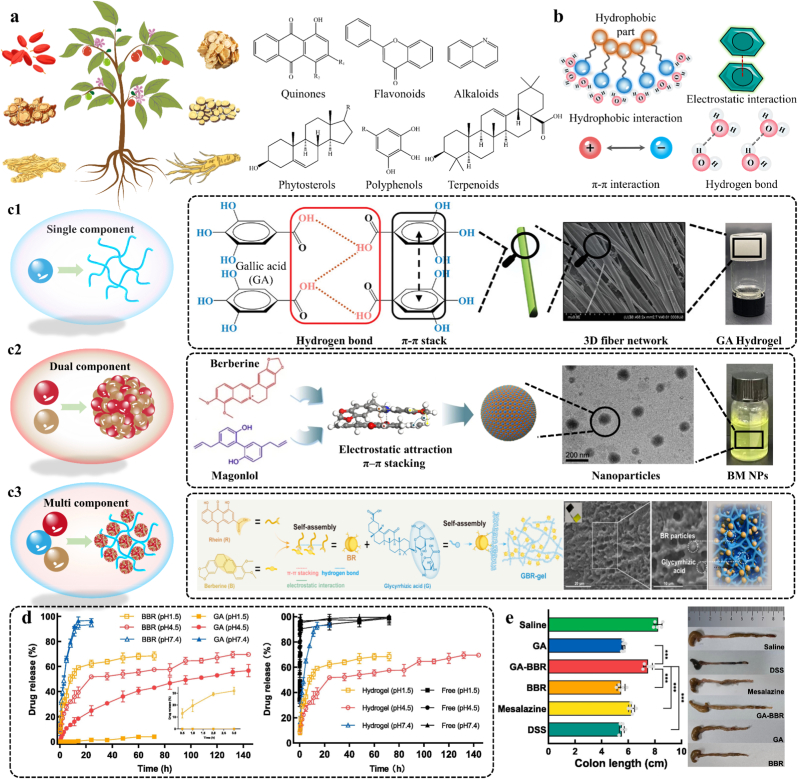
Natural Carrier-Free Assembled Materials-based DDS. **a)** Types of natural herbal components for the formation of supramolecular SAMs. **b)** Driving force for self-assembly of natural herbal components. **c1)** Single-component SAM: Ellagic acid forms a hydrogel rich in 3D fiber networks through hydrogen bonds and π-π stack. Adapted with permission [82]. Copyright 2022, John Wiley and Sons. **c2)** Dual-component SAM: Berberine and magnolol self-assemble to form nanoparticles through electrostatic attraction and π-π stacking. Adapted under the terms of the CC-BY license [83]. Copyright 2024, The Authors, published by Springer Nature. **c3)** Multicomponent SAM: Rhein, berberine and GA forms GBR-gel through hydrogen bond, π-π stacking and electrostatic interaction. Adapted with permission [84]. Copyright 2024, American Chemical Society. **d)** GA-BBR hydrogels enable pH-dependent sustained release. Adapted with permission [75]. Copyright 2025, Elsevier. **e)** The recovery of the length of the colon in colitis. Adapted with permission [75]. Copyright 2025, Elsevier.

Supramolecular SAMs derived from natural herbal components are classed as the nanomaterials, and we summarize types of natural herbal components that form SAMs in Fig. 5a. Active ingredients in natural herbs (such as polyphenols, alkaloids, terpenes, quinones) can serve as building blocks for supramolecular carrier-free self-assembly, with their intrinsic structural features as the core driver of programmable self-assembly. In supramolecular building block molecules, polar and non-polar groups determine hydrophilic/hydrophobic differences, aromatic rings govern π-π stacking interactions, hydrogen bond donor/acceptor sites mediate hydrogen bonding, and charge properties dictate electrostatic interactions. The design of their molecular structures aims to minimize lattice energy and maximize structural stability through intermolecular non-covalent interactions. The exchange and reorganization of building blocks in assemblies are governed by strict hydrogen bond donor/acceptor matching rules [73]. For example, the assembly of berberine (a hydrogen bond acceptor) and baicalin (a hydrogen bond donor) is governed by the "salt-cocrystal continuum" mechanism: the carboxyl group of baicalin forms a directional hydrogen bond network with the quaternary ammonium nitrogen atom of berberine, resulting in non-random aggregation [74]. This specific molecular recognition facilitates the formation of stable supramolecular synthons, which then assemble further into larger nanostructures.

These materials form through non-covalent interactions, including hydrogen bond, π-π stacking, hydrophobic forces, and electrostatic interaction (Fig. 5b). For example, glycyrrhizic acid (GA) and berberine (BBR) formed carrier-free hydrogels through hydrogen bond, π-π stacking and electrostatic interaction [75]. Hydrogen bonds provide directional stability in epigallocatechin gallate (EGCG)-curcumin (CUR) co-assemblies [76]. Hydrophobic interaction dominates curdlan-polyphenol systems, where the hydrophobicity of polyphenols dictates loading efficiency [77]. π-π stacking is critical in aromatic systems, such as berberine-rhein nanoparticles [78]. Electrostatic interaction enables charge-driven aggregation, as in cationic berberine and anionic cinnamic acid nanoparticles [79]. These forces program morphology: hydrophobic polyphenols favor the formation of nanoparticles, while amphiphilic molecules like GA and puerarin co-assemble into fibrous hydrogels [80].

Carrier-free drug delivery is a novel technology that delivers drugs directly to target cells or tissues without traditional carriers such as liposomes. It was hope to address critical limitations of traditional carriers, including carrier-associated toxicity, limited drug-loading capacity, complex manufacturing processes, and off-target accumulation. Its core design principle: rational selection of molecular components and assembly modes to program functions. Chen et al. reported that compared to a gel carrier group, natural free-carrier self-assembled diterpene nanoparticles significantly promote wound healing [81]. These carrier-free systems derived from natural small molecule compounds can be categorized into single-, dual-, and multi-component architectures. Single-component assemblies, such as gallic acid hydrogels, harness intrinsic molecular interactions to form stable structures without exogenous carriers to improve bioavailability (Fig. 5c1) [82]. Dual-component systems like berberine-magnolol (BM) co-assemblies utilize complementary interactions to enhance structural stability and bioactivity, as seen in improved colon-targeted delivery (Fig. 5c2) [83]. Multicomponent systems involve three or more components. A small-molecule hydrogel co-assembled by GA, BBR, and rhein allows prompt administration at brain injury sites to exert potent pharmacodynamic effects (Fig. 5c3) [84]. Carrier-free drug delivery strategies involve high drug-loading capacities to outperform traditional carrier systems. Moreover, many assemblies retain inherent bioactivity such as EGCG-CUR nanoparticles which exhibit reactive oxygen species (ROS) scavenging and anti-inflammatory effects and merge drug delivery with additional therapeutic functionalities [76]. In addition, these systems exhibit programmable dynamic architectures with high biocompatibility, stimuli-responsiveness, carrier-related toxicity avoidance, and simplified manufacturing processes. Environmental triggers such as pH or reactive oxygen species (ROS) enable dynamic responsiveness. For instance, celastrol-erianin nanoparticles disassemble in acidic tumor environments, while GA-BBR hydrogels achieve pH-dependent drug release (Fig. 5d) [75,85]. The core design of the GA-BBR co-assembled hydrogel lies in that GA confers pH-sensitive gelation properties that remain stable at pH 7.4 but dissolve at colonic pH, while BBR exerts potent anti-inflammatory effects. This hydrogel fulfills two key functions: first, it realizes prolonged retention in the inflamed colon via gel adhesion; second, it enables pH-triggered BBR release to exert localized therapeutic action. As shown in Fig. 5e, the GA-BBR hydrogel significantly reduces colonic inflammation-induced colon shortening by 2.85-fold compared with mesalazine, which directly addresses the critical clinical challenges of insufficient drug accumulation and suboptimal efficacy in inflammatory bowel disease [75]. Such stimuli-responsiveness not only enhances targeted delivery but also mitigates off-target toxicity, aligning with the “self-delivering” nanomedicine concept by allowing precise control over drug release kinetics.

Supramolecular SAMs from small molecule compounds offer a transformative approach by integrating natural bioactive properties with programmable nanostructures. Future advances may emerge from screening natural compounds and developing computational models for supramolecular behavior, thereby accelerating personalized nanomedicine and precision therapies. These carrier-free systems utilize non-covalent interactions to overcome limitations in drug stability, toxicity, and tissue-specific targeting, establishing a versatile platform for therapeutic innovation.

### 4.2 Component unit-based programmability

#### 4.2.1 Amphiphilic block copolymer micelles

**Fig. 6:**
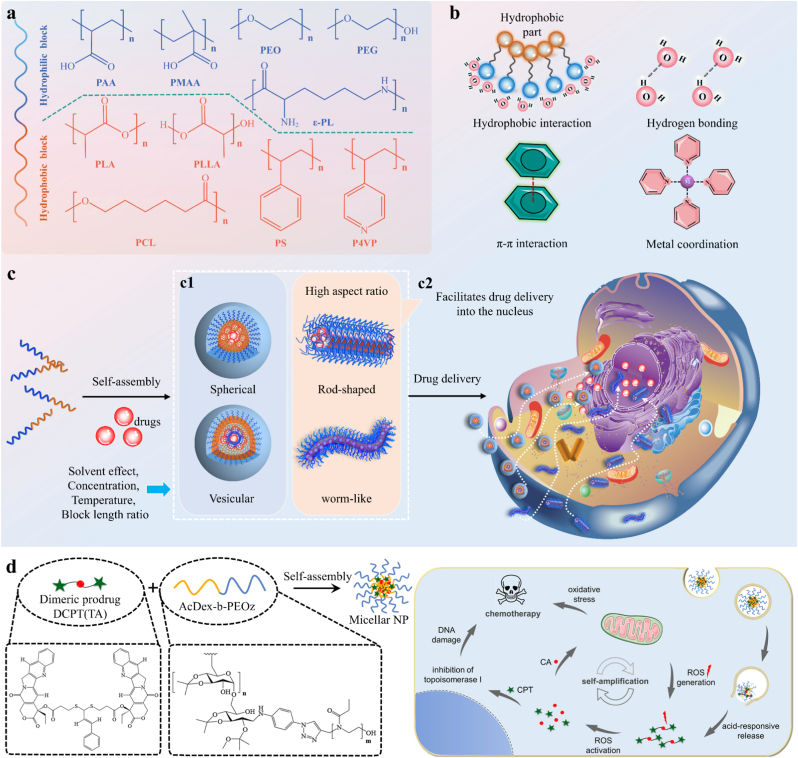
Amphiphilic block copolymer SAMs as DDS. **a)** Structural diagram and some common types of amphiphilic block copolymers. **b)** Driving force for self-assembly of amphiphilic block copolymer. **c1)** The self-assembly morphology of amphiphilic block copolymers and its influencing factors. **c2)** Cell uptake of different morphological SAMs. **d)** Self-assembled nanoparticles of amphiphilic block copolymers deliver dimeric prodrugs, with pH and ROS-responsive drug release in the intracellular environment being highlighted. Adapted with permission [99]. Copyright 2024, American Chemical Society.

Amphiphilic polymers self-assemble through non-covalent interactions to form multifunctional supramolecular materials with a core–shell architecture, where hydrophobic segments form the core and hydrophilic corona protects the exterior [86]. These materials typically form through self-assembly of block copolymers like poly(ethylene glycol)-poly(lactic acid) (PEG-PLA), with constituent blocks shown in Fig. 6a. During polymer self-assembly, hydrophobic interaction, hydrogen bond, π-π stacking, and metal-ligand coordination precisely control material stability (Fig. 6b). Studies show these materials exhibit programmable morphologies—spheres, worms, and vesicles—determined by hydrophilic-hydrophobic equilibrium, the kinetic equilibrium (nuclear crystallinity), and the thermodynamic equilibrium (solvent selectivity) [[87], [88], [89]]. Crystallization-driven self-assembly further enables programmable hierarchical nanostructures through kinetically controlled crystallization of hydrophobic blocks. Thermodynamically, entropy tends to promote the formation of patch-like structures by reducing interfacial energy between hydrophobic chain segments and solvent, while enthalpy dominates Janus structure formation through block incompatibility [90]. The controlled assembly of Janus structures shows excellent interfacial activity for stabilizing emulsions or polymer blends. In addition, programmable morphological transitions of these materials are influenced by solvent, concentration, temperature, and block length ratio (Fig. 6c1) [88]. CMCs decrease with increased proportion of hydrophobic blocks, and block copolymers form micelles through microphase separation, enhancing micelle stability [86].

Amphiphilic block copolymer micelles, with their core–shell architecture, serve as promising drug delivery carriers in pharmaceutical applications. Within DDS, these micelles effectively enhance the solubility and bioavailability of hydrophobic therapeutics. Feizpour et al. prepared amphiphilic diblock copolymer (PHPMA-b-PBAEM) micelles to encapsulate hydrophobic drug SN38 by single emulsification to improve solubility, stability, and targeting while reducing systemic toxicity [91]. The efficiency of payload encapsulation is governed by two factors: drug-micellar core compatibility and polymer concentration. These parameters dictate the encapsulation capacity and stability of drug-loaded micelles, thereby influencing their performance in vivo. Additionally, its performance in vivo is also influenced by the SAMs morphology. Liu et al. reported that at comparable volumes, high-aspect-ratio elliptical microgels are more readily enveloped by cell membranes than spherical particles, which remain partially enveloped on the membrane surface [92]. This finding indicates that anisotropic soft-matter nanoparticles are more susceptible to cellular phagocytosis, which is consistent with the results reported by Mitragotri et al.. [93] However, complete membrane envelopment of high-aspect-ratio nanoparticles requires a longer duration. Both spherical and rod-shaped gold nanoparticles can be internalized by mammalian cells, but the cellular uptake of rod-shaped gold nanoparticles takes significantly more time [94]. Hinde et al. found that the membrane penetration efficiency of differently shaped polymeric nanoparticles (e.g., micelles, vesicles, rods, and worm-like structures) is independent of particle morphology, which might be attributed to the variations in material compositions and surface properties [95]. Furthermore, nanoparticles of all tested shapes were able to escape endosomes and enter the cytoplasm. Notably, high-aspect-ratio nanoparticles (rods and worm-like structures) could traverse into the nucleus, whereas spherical counterparts (micelles and vesicles) failed to penetrate the nuclear membrane (Fig. 6c2). In conclusion, nanoparticle shape represents a critical programmable design parameter that is independent of size and surface chemistry. By modulating the balance between adhesion and internalization, it directly regulates cellular uptake efficiency and targeting precision [96,97].

A key advancement in this field lies in stimuli-responsive release mechanisms, which provide micellar systems with spatiotemporal control over drug release [98]. These mechanisms are triggered by physiological or pathological cues, including pH fluctuations, redox potential changes, ROS levels, or enzymatic activities. For example, a self-assembled system based on pH and ROS-responsive drug release is presented in Fig. 6d. The amphiphilic block copolymer (AcDex-b-PEOz) forms micelles encapsulating the dimeric prodrug DCPT. Upon entering the acidic tumor microenvironment (pH responsiveness), the micelle disassembles to release DCPT. Subsequently, intracellular esterases cleave DCPT to release active CPT, which induces ROS generation in mitochondria. This elevated ROS level further accelerates the degradation of the ROS-sensitive prodrug (ROS responsiveness), creating a self-amplifying oxidative stress loop for enhanced cytotoxicity. ROS-activatable prodrugs represent an approach where the intracellular oxidative stress is self-amplified, accelerating the activation of the encapsulated drug and enhancing therapeutic efficacy [99]. pH-responsive micelles exploit protonation/deprotonation of functional groups (e.g., poly(methacrylic acid)) to trigger payload release in gastrointestinal environments [55]. Redox-sensitive systems rely on ferrocene oxidation, where cellular redox changes cause micelle disassembly and drug release [100]. These designs show how micellar carriers can be engineered for targeted therapy. Amphiphilic block copolymer micelles, combining self-assembly with stimuli responsiveness, represent a transformative paradigm in drug delivery for therapeutic cargo transport.

#### 4.2.2 Ionic liquids and deep eutectic solvents

**Fig. 7:**
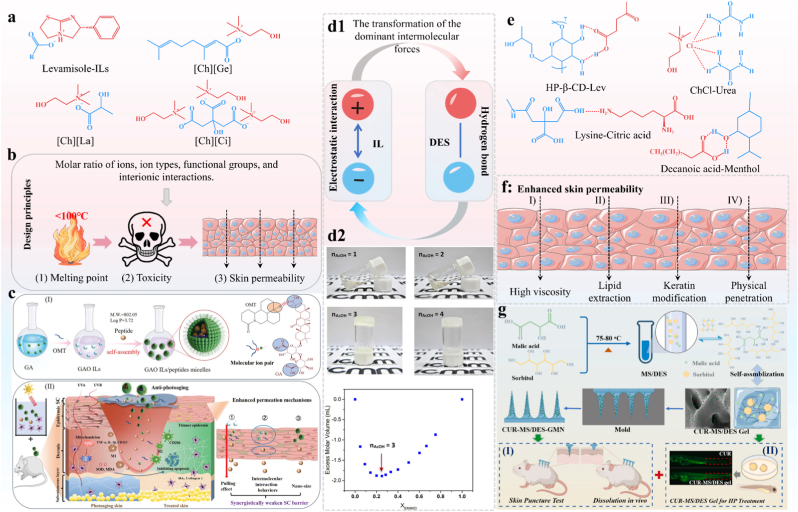
ILs and DESs-based DDS. **a)** The structures of several common ILs. **b)** The design principles of ILs. **c)** Schematic of the synthesis, permeation, and anti-photoaging effects of GAO/PAL-4-SM. Adapted under the terms of the CC-BY license [107]. Copyright 2025, The Authors, published by John Wiley and Sons. **d1)** The transformation between ILs and DESs. **d2)** the transition from 1-ethyl-3-methylimidazolium chloride (EMIMCl) to EMIMCl·nAcOH-based DESs. Adapted with permission [110] Copyright 2022, American Chemical Society. **e)** The structures of several common ILs. **f)** The mechanism of DES enhancing transdermal permeability. **g)** Schematic of the formation process for CUR-MS/DES-GMN and its potential application as a TDDS for HP treatment. Adapted under the terms of the CC-BY license [113]. Copyright 2024, The Authors, published by Elsevier.

ILs are organic salts with melting points below 100 °C, synthesized from biocompatible cations and anions, often derived from small molecule compounds to enhance the solubility, permeability, and therapeutic efficacy of active pharmaceutical ingredients (APIs). Some structures of ILs are shown in Fig. 7a. IL-based SAMs are governed by electrostatic interaction between oppositely charged ions, which stabilize the structure. However, the strong hydrogen bonds and van der Waals forces between ions influence the interionic interaction strength, modulating the lattice energy of ILs [101]. This highlights the critical role of force balance in maintaining ILs stability. Unlike carrier-free DDSs that self-assemble from bioactive small molecule compounds of natural origin, IL-based systems offer tunable physicochemical properties through anion/cation selection, enabling control over drug loading and biological barrier interactions. Their advantages include enhanced biocompatibility, simple preparation, and the ability to integrate multiple functions (e.g., permeation enhancer, solvent, stabilizer) in a single platform.

APIs are incorporated as structural components into ILs for drug delivery. For example, Moshikur et al. synthesized ILs by combining hydrophilic drugs such as lidocaine, imipramine, and levamisole hydrochloride with fatty acid salts, enhancing transdermal penetration of hydrophilic drugs [56]. Numerous recent studies have shown that IL-based SAMs deliver diverse APIs, including small molecule compounds [102], peptides [103], and proteins [104], showing potential in transdermal, oral, and mucosal drug delivery. In transdermal delivery, ILs improve drug permeation by reducing mucus viscosity and opening tight junctions. Specifically, ILs fluidize stratum corneum (SC) lipids, induce phase transitions, and create transient pores, as shown by solid-state NMR and microscopy. This dual mechanism—lipid disordering combined with enhanced drug partitioning—enables efficient macromolecule transport [[103], [104], [105]]. Chen et al. identify key design parameters for transdermal ILs as melting point, skin permeability, and toxicity, which depend on ion ratio, types, functional groups, and interionic interactions (Fig. 7b) [106]. Wang et al. constructed a transdermal delivery system for signal peptides by using GA and oxymatrine (OMT) IL, where micelles penetrate the skin barrier, regulate immune phenotype, act as antioxidants, inhibit apoptosis, and promote matrix synthesis, offering a strategy of “carrier design-transdermal enhancement-target regulation” for anti-skin photoaging (Fig. 7c) [107]. Additionally, other design strategies focus on tailoring anion/cation hydrophobicity and molecular weight to balance permeation enhancement and biocompatibility. The alkyl chain length and saturation of fatty acids influence SC lipid fluidization, with oleate-based ILs showing superior transdermal ability compared to laurate or stearate variants [56].

The formation of DESs-based SAMs is primarily driven by hydrogen bond interactions. The cyclodextrin-levulinic acid DES exhibits a structural backbone of dense hydrogen bonds [108]. Differently, the hydrogen bond pattern in oxymatrine-fatty acid DES forms a “salt-cocrystal continuum” charge-assisted interactions [109]. DESs have become preferred solvents in many processes, similarly to ILs. Despite the main interactions differing between DESs (H-bond-type) and ILs (electrostatic-type), both solvents share similar physiochemical properties. So, DESs often used as an alternative to ILs. The transition from ILs to DESs is achieved by strategically modifying the dominant intermolecular interactions through the addition of hydrogen bond donors (HBDs) (Fig. 7d1). AcOH, a hydrogen bond donor (HBD), added to ionic liquid EMIMCl disrupts the electrostatic network between EMIM^+^ and Cl^−^; Cl^−^ preferentially forms H-bonds with AcOH, shifting the dominant interaction from electrostatic (ILs state) to H-bonding (DESs state). Zhang et al. found only mixtures with nAcOH = 1 and 2 remained liquid at −78 °C (IL state). By measuring density at 85 °C to calculate excess molar volume (*V*^*E*^), they observed negative *V*^*E*^ deviation (mixture compression). In non-ideal H-bonded (HB) mixtures, negative *V*^*E*^ arises from molecular embedding into each other's HB networks, forming stronger and more hybrid HB networks. The maximum deviation occurred at χEMIMCl≈0.23 (i.e., EMIMCl·AcOH with nAcOH = 3), thus it was identified this mixture as the eutectic state. (Fig. 7d2) [110]. DESs form through mixing HBA (e.g., quaternary ammonium salt) and HBD (e.g., urea, carboxylic acid, *etc.*) in specific ratios to create supramolecular complexes with lower melting points than individual components. While sharing characteristics like low volatility with ILs, DESs feature nonionic interactions and broader compositional diversity [110]. DESs overcome the cost and toxicity limitations of ILs through component compounding while maintaining tunability, making them suitable for pharmaceutical applications [111]. Some structures of DESs are shown in Fig. 7e.

DESs function as solubilizing agents that enhance dissolution of poorly soluble drugs and facilitate delivery. For example, hydrophobic drugs like resveratrol and curcumin show improved solubility when dissolved in DES [112]. DES are widely used in transdermal delivery systems due to their ability to enhance skin permeation through multiple mechanisms (Fig. 7f): Ⅰ) High viscosity: DES's elevated viscosity improves skin retention and prolongs drug-skin contact time. Ⅱ) Lipid extraction: DESs extract intercellular lipids from the stratum corneum (SC), fluidizing its “brick” structure and reducing barrier integrity. Ⅲ) Keratin modification: DESs' hydrogen bonds alter keratin's secondary structure in the SC. Ⅳ) Physical penetration enhancement: Self-assembled gel microneedles with DESs expand transdermal strategies. For instance, a malic acid-sorbitol DESs self-assembles into a three-dimensional gel network via hydrogen bonds, providing mechanical strength to penetrate skin and enhance curcumin release (Fig. 7g) [113]. The integration of DESs with advanced formulations demonstrates their versatility in addressing drug delivery challenges and offering innovative solutions to overcome complex bioavailability barriers.

#### 4.2.3 Self-assembling peptides materials

**Fig. 8:**
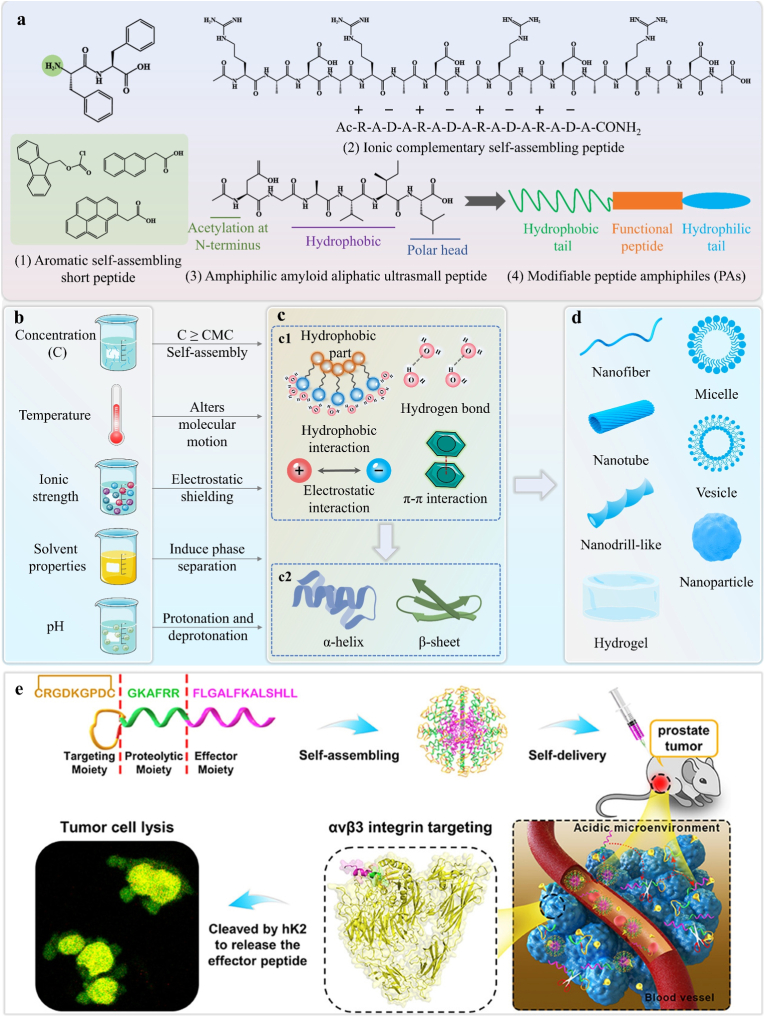
Self-assembling peptide SAMs-based DDS. **a)** The four main peptide self-assembly systems and representative examples: Aromatic self-assembling peptides (FF and common N-terminal aromatic substituent groups: naphthalene, Fmoc, and pyrene); ionic complementary self-assembling peptides (RADA16); amyloid aliphatic self-assembling ultrasmall peptides (AcLD6: Ac-LIVAGD); and modifiable peptide amphiphiles (PAs). (Fmoc: Fluorenylmethyloxycarbonyl). Adapted under the terms of the CC-BY license [126]. Copyright 2022, The Authors, published by John Wiley and Sons. **b)** Environmental factors influencing the self-assembly process of peptides. **c1)** The driving force of peptide self-assembly. **c2)** The secondary structure of peptide self-assembly. **d)** The characteristic morphology formed by peptide self-assembly. **e)** Schematic of the design of a targeted-proteolytic-effector-integrated peptide-based self-assembling material DDS. Reproduction with permission [119]. Copyright 2022, American Chemical Society.

Peptide chains, composed of amino acids, act as constituent units for proteins, but not all peptide units have the same self-assembly ability. The design of self-assembling peptides depends primarily on peptide sequence, as different amino acid sequences form distinct conformations that influence their interaction patterns and final self-assembled architectures. Shuguang Zhang at MIT elaborated the design principles of self-assembling peptide sequences, showing that amino acids with alternating hydrophobic/hydrophilic sides or complementary ionic sides based on the orientation of positive and negative charges, form the foundational blocks of self-assembling peptides [29,114]. Furthermore, Fig. 8a illustrates the types of peptides exhibiting self-assembly behavior, providing a useful reference for programming self-assembling peptide sequences. Peptide-based SAMs are bioinspired nanostructures formed through programmable spontaneous organization of peptide building blocks. This process is driven by non-covalent interactions, such as hydrogen bond, hydrophobic effects, electrostatic interaction, and π-π stacking, which are dependent on differences in amino acid sequences (Fig. 8c1). These materials self-assemble into well-ordered nanostructures (Fig. 8d) such as micelles [115], nanofibers [116], nanodrill-like [117], nanotubes [118], vesicles [118], hydrogel, and nanoparticles [119], exhibiting tailorable mechanical properties, biocompatibility, and biodegradability. Ultimately, the programmable design of peptide sequences determines the assembly dynamics and morphology of the nanostructures. Peptide self-assembly is driven by a balance of non-covalent interactions between amino acid residues, which play key roles in dictating nanostructures.

A hierarchical assembly mechanism is clarified where hydrophobic interactions and hydrogen bonding play distinct but cooperative roles in determining peptide morphology. The hydrophobic effect, primarily arising from aromatic residues (e.g., Phenylalanine in FF peptides), acts as the primary thermodynamic driver to initiate molecular aggregation from the aqueous phase, reducing the interfacial energy [117]. However, it is the intermolecular hydrogen bonding (specifically between the carbonyl oxygen and amide hydrogen of the peptide backbone) that acts as the architect, directing the aggregated peptides to lock into specific secondary structures, such as β-sheets. Secondary structures play a critical role in determining self-assembled morphologies (Fig. 8c2). β-sheets are strongly associated with formation of nanofibers, while α‐helices form nanoparticles [119]. Research by N. Ashwanikumar et al. showed that transitions between secondary structures depend on the number of consecutive N-terminal phenylalanine (Phe) residues: even numbers of consecutive Phe residues favor α-helices, while odd numbers promote β-sheets, influencing the final morphology [117]. Such structural transitions generate a directional force that promotes longitudinal stacking, driving the transformation from amorphous aggregates into high-aspect-ratio nanofibers or nanotubes. Accordingly, this sequence-dependent design principle enables the programmable fabrication of nanostructures tailored to address clinical challenges. A design choice uses peptides with odd consecutive N-terminal Phe residues assembling high-aspect-ratio nanofibers whose elongated shape navigates tumor stroma better than spheres. Furthermore, π-π stacking between aromatic rings provides auxiliary stabilization for the assembled scaffold, enhancing the mechanical stiffness of the resulting nanomaterials. In summary, hydrophobic interaction initiates aggregation, hydrogen bonds and electrostatic interaction refine and stabilize the structure, collectively programming diverse programmable nanostructure formation.

Environmental factors, as external modulators, also critically influence peptide self-assembly by modulating non-covalent interactions and secondary structures (Fig. 8b). Key parameters include concentration, temperature, pH, ionic strength, and solvent properties. Below critical assembly concentration (CAC), peptides remain disordered; exceeding CAC triggers β-sheet formation and nanofiber assembly [116]. Thermal cycling (heating/cooling) alters molecular motion, enabling the transition of weak polypeptide gels into nanofibers [120]. Solvent gradients (e.g., acetone-water mixtures) induce phase separation, promoting β-sheet formation and hollow capsule assembly [121]. Under physiological ionic strength, MAX8 adopts a β-hairpin conformation, forming bilayer nanofibers, while Ca^2+^ shields electrostatic repulsion between fibers, enabling gelation via 3D entanglement [54,122]. The impact of pH is particularly profound. At low pH (≤9), arginine is protonated, driving α-helix formation and nanotube assembly. At a high pH (≥12), arginine is deprotonated, causing α-helices to form vesicles [118]. In another study, pH regulated the reversible morphological transition of polypeptides between nanofibers and nanoparticles. The pH-dependent protonation state of histidine residues governs charge variations, modulating intermolecular interactions and driving structural switching [123]. In conclusion, environmental factors control programmable self-assembly outcomes through noncovalent forces and secondary structures. Effective peptide design requires sequence optimization with environmental parameter control.

Peptide-based SAMs have shown remarkable functionality and potential in drug delivery. Previous sections covered peptide amino acid sequence design, and bottom-up design strategies for self-assembling peptides were explored by Kaygisiz et al., [124] providing a foundation for programmable peptide-based SAMs. Furthermore, design of multifunctional peptide-based SAMs remains a research focus. Altunbas et al. conjugated dexamethasone to a self-assembling peptide via hydrazone bonds, creating a nanofiber hydrogel with sustained-release and anti-inflammatory functions [54]. The hydrazone bonds enable pH-responsive drug release. Researchers can develop other responsive systems, including temperature-, enzyme-, ROS-, and redox-responsive systems. Pei et al. designed LASAP1, a self-assembling anticancer peptide capable of hK2 enzyme-responsive cleavage by prostate cancer cells [119]. Recent studies show increased multifunctional integration in SAMs design (Fig. 8e). To address the core clinical challenges of poor targeting, high toxicity, and low efficacy in prostate cancer therapy, Tian et al. proposed a targeting-hydrolysis-transformation strategy, integrating therapeutic peptide delivery, targeted transport, enzyme-responsive hydrolysis, and redox-responsive release [125]. The targeting segment CRGDKGPDC binds to αvβ3 integrin overexpressed on tumor/vascular surfaces for active targeting; the enzymatic cleavage segment GKAFRR contains a specific cleavage site for hK2; and the effector segment FLGALFKALSHLL exerts direct tumoricidal activity. These modules self-assemble into nanocarriers, delivered intravenously via the synergy of active targeting and passive EPR accumulation. The hK2 enzyme in the tumor microenvironment then triggers cleavage of the enzymatic segment, releasing the effector peptide to induce tumor lysis. This integrated modular design resolves core predicaments in clinical prostate cancer therapy through modular synergy. This programmable design has significantly advanced innovation in DDS. Despite these advances, challenges in scaling production and ensuring stability require further exploration of sequence-structure-function relationships. In conclusion, supramolecular self-assembling peptides offer a transformative platform for drug delivery, combining programmable design and stimuli-responsive behavior to address therapeutic needs.

#### 4.2.4 Polysaccharide assembled materials

**Fig. 9:**
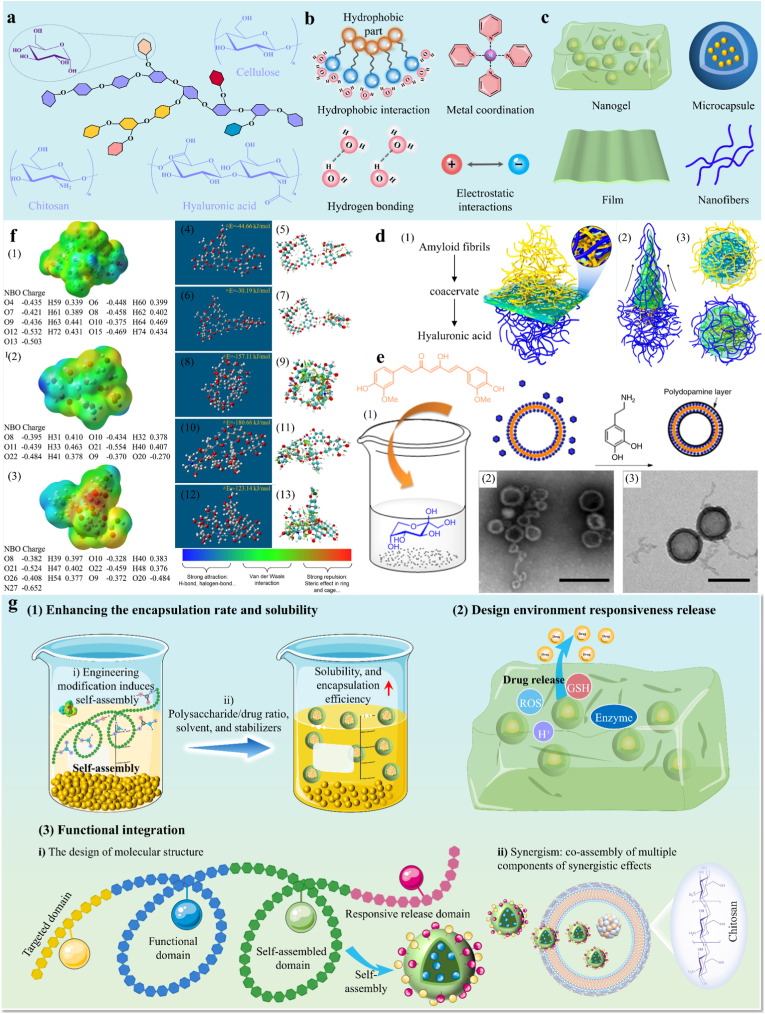
Natural polysaccharides SAMs-based DDS. **a)** Schematic and typical structure of natural polysaccharides. **b)** Driving force for self-assembly of polysaccharides. **c)** The product of polysaccharide supramolecular self-assembly. **d)** Schematic of AF and HA interfacial coacervation for developing advanced materials. (1) films, (2) fibers and (3) capsules. Adapted under the terms of the CC-BY license [129]. Copyright 2023, The Authors, published by Springer Nature. e) Formation of curcumin-based capsules induced by the presence of sugar and their stabilization using dopamine. Adapted under the terms of the CC-BY license [138]. Copyright 2019, The Authors, published by Springer Nature. **f)** DFT theoretical calculations of the electrostatic potential and charge distribution between the carrier (FAIP and QFAIP) and the drug (HES). Adapted with permission [132]. Copyright 2024, Elsevier. **g)** The design strategy of polysaccharide SAMs DDS. (1) Enhancing the encapsulation rate and the solubility; (2) Design environment responsiveness release; (3) Functional integration.

Saccharides are multiple hydroxyl organic compounds. Disaccharides, oligosaccharides, and polysaccharides consist of monosaccharide units linked through glycosidic bonds. The structural complexity of saccharides stems from monosaccharide types, glycosidic bond configuration, unit arrangement, polymerization degree, and branched chain diversity. This determines their advanced chain conformations through supramolecular self-assembly. Natural polysaccharides are biopolymers derived from plants, animals, algae, and microorganisms, constituting over 90 % of total carbohydrates in nature as green biological raw materials [127]. The schematic and common structures of polysaccharides are shown in Fig. 9a. Natural polysaccharides-based SAMs form through hydrophobic interactions, hydrogen bonds, electrostatic interactions, and metal-ligand coordinations (Fig. 9b). Hydrophobic regions of polysaccharides tend to reduce their contact area with polar solvents through hydrophobic interaction, and aggregate to form a hydrophobic core. While charged polysaccharides form multilayered structures through electrostatic interaction which govern molecular adsorption and arrangement, and participate in the formation of pH-responsive dynamics. Polyhydroxy groups create dynamic hydrogen bond networks for molecular alignment and participate in metal coordination to form rigid network skeletons. These driving forces enhance structural stability, enabling polysaccharides to self-organise into ordered nanostructures with tunable properties, forming programmable hierarchical architectures like nanogels, microcapsules, fibers, and films (Fig. 9c). Furthermore, the self-assembly mechanisms are dynamic and reversible, allowing structural adaptability. For instance, electrostatic interaction between cationic trimethyl chitosan (TMC) and anionic salecan forms nanogels. Under acidic conditions, the carboxyl groups of salecan are protonated, reducing charge density and enhancing intra-/intermolecular hydrogen bonds to form a compact and reversible network. When pH shifts neutral, the change in the charge interaction causes the network to loosen, triggering drug release [128]. Recent studies of amyloid-polysaccharide coacervates for gastric ulcer protection have demonstrated that the programmable morphological transformation through interfacial self-assembly (Fig. 9d) [129]. Interfacial coacervation between Amyloid fibers (AF) and hyaluronic acid (HA) solutions enables flexible control of self-assembly morphology, facilitating efficient preparation of three material types: films (via layered contact), continuous fibers (via extrusion spinning), and microcapsules (via solution injection).

Natural polysaccharides-based SAMs confer the characteristics of biocompatibility and biodegradability due to their natural source, and compared to CD-based SAMs, polysaccharides offer enhanced multifunctionality with intrinsic antioxidant or anti-inflammatory activity. Thus, polysaccharides have attracted increasing attention as drug delivery carriers. Fructose and curcumin self-assemble to form vesicles (Fig. 9e), which can be used for the delivery of curcumin. Drug-loading mechanisms of natural polysaccharides-based SAMs rely on physical entrapment, electrostatic adsorption, or covalent conjugation. For example, polysaccharide (FS60)-curcuminoid aggregates encapsulate curcumin via hydrophobic interaction, while pathogen-mimicking mannan nano vaccines load antigens through electrostatic and hydrophobic interaction [130,131]. In recent studies, molecular-level interactions between polysaccharides and active pharmaceutical ingredients have been visually analyzed by density functional theory (DFT) computational models (Fig. 9f) [132]. The development of computational models provides guidance for the stability of binding between drugs and carriers, and provides a theoretical basis for the programmable design of SAMs.

The design of polysaccharide-based SAMs DDSs focuses on optimizing encapsulation efficiency, environmental responsiveness, and versatility (Fig. 9g). Ⅰ) Enhance drug encapsulation efficiency and solubility. Using DFT computational modelling, polysaccharides are modified with functional groups (hydroxyl, carboxyl, and quaternary ammonium) to enhance hydrogen bonds, electrostatic interaction, and hydrophobic interaction with drugs. The process is optimized by adjusting polysaccharide-drug ratios, selecting appropriate solvents (ethanol/water systems, choline–diethanolamine DES systems), and incorporating stabilizers. This systematic optimization enables precise control of the self-assembly process, significantly improving both the encapsulation efficiency and solubility of hydrophobic drugs [130,132]. Ⅱ) Design environment-responsive release for improved bioavailability. Environmental factors like pH, ionic strength, and enzyme activity regulate assembly and drug release. pH-responsive systems modulate polysaccharide ionization states. For example, Salecan-TMC hydrogels restrict drug release in acidic conditions via carboxyl protonation, while neutral pH induces ionization and network swelling for enhanced intestinal release [128]. Redox-responsive systems use elevated ROS levels to cleave polysaccharide-metal coordination bonds in Shik-Fe@GUM for antioxidant release [133]. Enzymatically responsive designs use specific degradation, shown by hyaluronidase-cleavable nanogels releasing cisplatin in lysosomes while chloroquine enhances efficacy [134]. Ⅲ) Functional integration for application potential. Molecular-level modifications (e.g., sulfonation, carboxymethylation) through functional groups or crosslinking (Ca^2+^) provide targeting capabilities and environmental responsiveness, or mechanical reinforcement properties. Compositionally, combining multiple polysaccharides (e.g., pectin-chitosan) or forming polysaccharide-polyphenol/protein complexes enables functional complementarity [[135], [136], [137]]. In summary, supramolecular polysaccharide systems offer a versatile platform for drug delivery, combining natural origin with programmable assembly mechanisms and multifunctionality.

### 4.3 Precise rule-based programmability

#### 4.3.1 Metal-organic framework

**Fig. 10:**
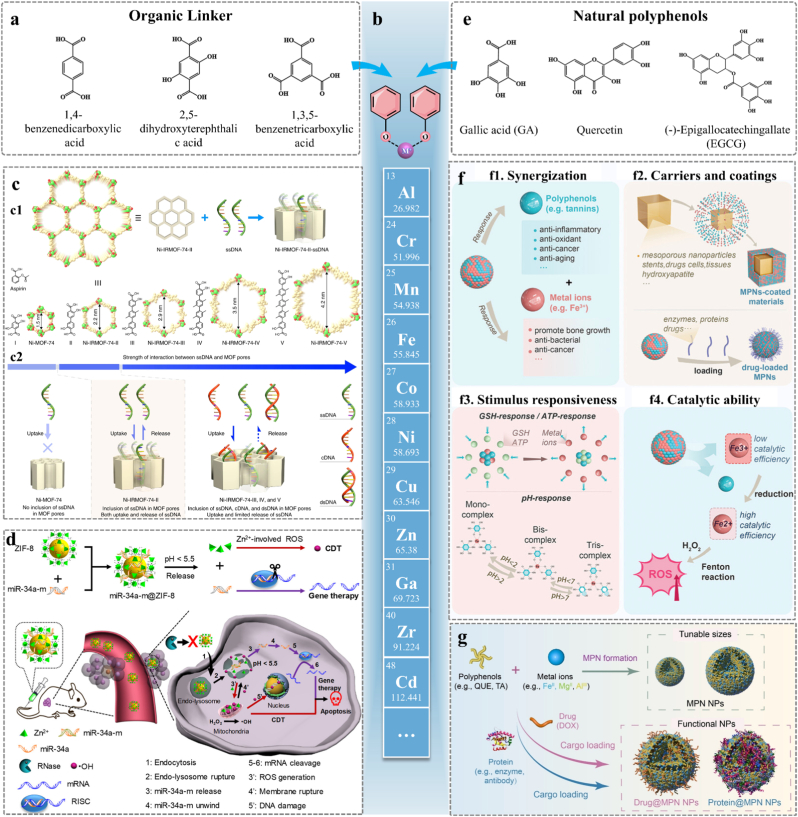
Metal-coordination-mediated SAMs DDS. **a)** The structures of several common organic ligand linkers. **b)** Selected range of metal ions that have been reported to construct MOFs. **c1)** The organic linker precisely controls the pore size of the MOF. Adapted under the terms of the CC-BY license [143]. Copyright 2018, The Authors, published by Springer Nature. **c2)** Gradual increase of interaction between ssDNA and MOFs as the pore size of MOF extended progressively. Adapted under the terms of the CC-BY license [143]. Copyright 2018, The Authors, published by Springer Nature. **d)** Schematic of miR-34a-m@ZIF-8 Composite System for Synergetic Gene/Chemodynamic Therapy. Adapted with permission [148]. Copyright 2021, American Chemical Society. **e)** The structures of several common natural polyphenol for MPNs. **f)** Schematic representation of the properties of MPNs. Adapted under the terms of the CC-BY license [52]. Copyright 2024, The Authors, published by John Wiley and Sons. **g)** Schematic of the direct assembly of MPN NPs and cargo-loaded MPN NPs. Adapted under the terms of the CC-BY license [149]. Copyright 2023, The Authors, published by John Wiley and Sons.

MOFs are hybrid porous materials constructed through coordination self-assembly of metal ions/clusters with organic ligands. Driven by coordination bonds, metal ions self-assemble with imidazole or carboxylic acids, forming rigid crystal structures. Common organic ligand linkers are illustrated in Fig. 10a. Based on metal ions, common types include Zn-MOFs, Zr-MOFs, Cu-MOFs, Fe-MOFs, and Ca-MOFs [139]. The metal ions used to construct metal-coordination SAMs are shown in Fig. 10b. The coordination flexibility of Cu(I)-isopolymolybdate-based MOFs and zinc-ions-2-methylimidazole networks (ZIF-8) enables structural adaptability [22,140]. The self-assembly process of MOFs is essentially dominated by metal-ligand coordination bonds, which possess strict geometric directionality due to the involvement of metal d-orbitals. In the assembly kinetics, metal nodes (e.g., Zn^2+^, Co^2+^) act as rigid vertices, while organic ligands serve as directional linkers. Coordination bonds drive the nucleation process through the rearrangement of amorphous particles via metastable phases by overcoming the competition between interfacial energy and bulk energy. With the continuous formation of coordination bonds, small crystallites dissolve, and their dissociated monomers deposit directionally on the surface of large crystals, realizing the expansion of the crystal framework through crystallographic chain growth or step growth mechanisms [141]. In summary, the highly directional bonding network constructed by coordination bonds can force molecules to arrange orderly according to the predetermined topology, thereby determining the long-range order of the MOF lattice. With crystalline frameworks, high surface areas, and adjustable pore functionalities, MOFs serve as ideal candidates for drug delivery applications. The ligand length determined the distance between metal nodes, affecting MOF pore size (Fig. 10c1). Yuan et al. demonstrated that MOF unit cell dimension, surface area, and pore size were precisely controlled by adopting different linker sets and ratios [142]. Similarly, Peng et al. confirmed this finding [143]. Interestingly, the interaction between ssDNA and MOFs gradually increases with expanding pore size of MOF (Fig. 10c2). Based on the previous research findings, Łuczak et al. conducted a systematic review and analysis on the morphology regulation strategies of MOFs. The regulation mechanisms of MOF morphology can be summarized into four categories: (ⅰ) coordination modulation mechanism; (ⅱ) protonation/deprotonation mechanism; (ⅲ) surfactants/capping agents' modulation mechanism (ⅳ) energy supply mode regulation mechanism [144]. Notably, the microscopic morphological characteristics of MOFs are closely correlated with, even play a decisive role in, their application performance as drug carriers. Specifically, high specific surface area and porosity can provide abundant drug adsorption sites; excellent crystallinity is capable of reducing the risk of drug leakage and improving their stability during in vivo circulation; smaller particle sizes can accelerate the diffusion rate of drugs in biological media; anisotropic morphologies (e.g., rod-like or sheet-like structures); are more prone to be internalized by target cells, thereby enhancing the targeting efficiency of drug delivery.

The design of MOFs involves selecting biocompatible metal nodes (e.g., Zn^2+^) and functional ligands to enhance stability and bioactivity. Consequently, MOFs-based DDS demonstrate exceptional potential due to their biocompatibility and multifunctionality. Wang et al. developed a biocompatible ZIF-8-based nanoplatform where surface modifications enabled photosensitizers to circulate as small nanoparticles (∼50 nm) for tumor targeting [145]. Inside tumors, glutathione reduced disulfide bonds trigger self-assembly, forming large aggregates (>200 nm), enhancing drug retention. The highly porous ZIF-8 structure facilitated oxygen transport and minimized water-induced quenching, boosting singlet oxygen generation for photodynamic therapy with low toxicity and improved antitumor efficacy. Drug incorporation into MOFs relied on pore embedding, surface attachment, covalent binding, in situ encapsulation during synthesis, and formation of bio-MOFs. Among these, pore encapsulation is the most commonly used method. In current research, glabridin and polypyrrole nanoparticles act as “seeds”, with metal ions and ligands self-assembling on surfaces to form shell structures, enabling in-situ encapsulation within the pores [140,146]. The reversibility of coordination bonds enables stimuli-responsive behavior, including temperature, pH- or light-triggered drug release [147]. For example, acid-labile Zn–N bonds of ZIF-8 enable pH-responsive degradation in lysosomes, releasing therapeutics like miR-34a mimics while generating ROS for synergistic chemodynamic therapy (Fig. 10d) [148]. These systems demonstrate coordination-directed assembly and noncovalent interactions’ synergy, enabling precise spatiotemporal control over drug delivery. By leveraging dynamic supramolecular interactions, MOFs bridge the gap between structural precision and functional adaptability for next-generation smart therapeutics.

#### 4.3.2 Metal-phenolic networks

MPNs are a class of supramolecular materials formed through coordination of polyphenols with metal ions. Polyphenols (Fig. 10e) self-assemble with metal ions to form hierarchical nanostructures with tunable physicochemical properties. MOFs are characterized by their long-range ordered crystallinity and rigid porous frameworks, which are driven by strong, directional coordination bonds, thus creating permanent well-defined pores suitable for guest molecule encapsulation. However, unlike MOFs, MPNs possess amorphous networks with dynamic adaptability, enabling stimuli-responsive drug release and structural reconfiguration under physiological conditions [61]. The absence of crystallinity endows MPNs with a unique conformal coating capability, allowing them to conform to the surfaces of various substrates regardless of their morphologies. While MOFs excel in size-selective loading due to their rigid porosity, MPNs offer superior interfacial adaptability for surface engineering, thereby enabling mimicry of biological interfaces while circumventing the complexity and toxicity associated with MOFs [61,149]. Furthermore, the self-assembly process of MPNs features high modularity and spatiotemporal controllability. By virtue of a modular assembly strategy, Guo et al. revealed the mechanism underlying the transformation of diverse building blocks into Lego-like superstructures via the interfacial anchoring between polyphenols and substrates, as well as metal-mediated crosslinking. Driven by π-π interactions and dipole-dipole forces, the formation of hierarchical architectures is facilitated, thus enabling controllable self-assembly at the interfaces of cells, nanoparticles and other substrates to construct core-satellite, hollow or layered superstructures [150,151]. The hydroxyl groups in polyphenol molecules participate in the formation of hydrogen bonds, while π-π stacking and hydrophobic interactions between aromatic rings further enhance intermolecular forces and improve the overall stability. Such interfacial interactions also endow the system with light or pH responsiveness, which enhances the adaptability of the self-assembly process. In the context of MPN assembly for cell surface engineering, the reversibility of non-covalent interactions allows the rapid construction of cell-independent functionalized biohybrids [152].

As drug delivery platforms, MPNs excel due to their unique advantages, which align with their dynamic structural features. MPNs offer high drug-loading capacity through π-π interactions, hydrophobic encapsulation, or metal ion coordination (Fig. 10f2). Their dynamic bonds enable responsive release to pH, GSH, ATP, and ROS (Fig. 10f3 and Fig. 10f4), enhancing their utility in adaptive DDS [153,154]. MPN design emphasizes modularity through selecting bioactive polyphenols with specific bioactivities (e.g., anti-inflammatory tannic acid and honokiol, or antioxidant gallic acid) and pairing them with functional metal ions for photosensitizer (Fe^3+^), imaging (Gd^3+^), or catalytic (Pt^4+^) specific roles (Fig. 10f1) [61,153,155]. For example, Pt^4+^-honokiol nano-micelles utilize both chelation and hydrophobic interactions, demonstrating antibacterial activity and renal protection, highlighting the dual utility of polyphenol bioactivity and metal ion functionality [156]. Loading exogenous cargos further functionalizes MPNs (Fig. 10g) [149]. These versatilities extend to therapeutic synergy, as demonstrated in Fe^3+^-rosmarinic acid nanocomposites, which induce ferroptosis via Fenton reactivity while depleting GSH, integrating enzyme-mimetic activity with redox-responsive drug release for enhanced tumor therapy [154]. Guo et al. systematically summarized the roles of MPNs in advanced therapeutics, including intracellular energy conversion, immunotherapy, and targeted therapy, and highlighted their distinct advantages particularly in enhancing cell viability and targeting specificity [[157], [158], [159]]. MPNs integrate the principles of coordination chemistry, the merits of targeted and stimulus-responsive delivery, and biological adaptability, thus emerging as a multifunctional alternative to MOFs. The applications demonstrated in these studies fully validate the value of MPNs in personalized medicine and precision therapy.

#### 4.3.3 DNA origami

**Fig. 11:**
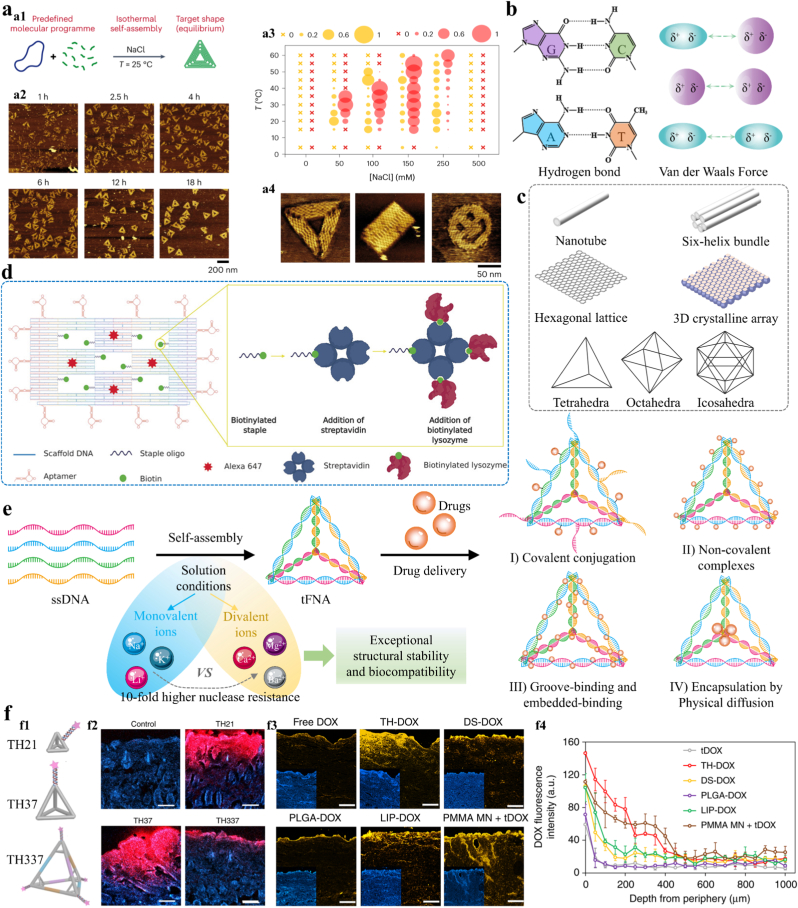
DNA origami SAMs for DDS. **a)** Isothermal self-assembly of user-defined DNA origamis in a magnesium-free NaCl buffer. Adapted under the terms of the CC-BY license [160]. Copyright 2023, The Authors, published by Springer Nature. b) The driving force of DNA origami. **c)** The typical nanostructures formed by DNA origami. **d)** Schematic illustration of DNA origami-based nanocarrier for targeted delivery of antibacterial agents. Adapted under the terms of the CC-BY license [166]. Copyright 2020, The Authors, published by John Wiley and Sons. **e)** Schematic of the construction strategy for the TFNAs DDS. **f)** Comparison of the transdermal delivery capabilities of TFNA in three different sizes. Adapted under the terms of the CC-BY license [174]. Copyright 2019, The Authors, published by Springer Nature.

In the evolution of life's origin, complementary base pairing serves as a fundamental mechanism for molecular self-assembly, driving nucleic acid monomers to form ordered structures encoding genetic information. This recognition system, evolved over billions of years, represents the natural oldest supramolecular self-assembly code. The emergence of DNA origami technology represents the precise decoding and innovative rewriting of natural code. As a cornerstone of structural DNA nanotechnology, it embodies a sophisticated form of supramolecular self-assembly driven by programmable Watson-Crick base pairing [32]. This technique allows precise folding of long scaffold DNA strands into two-dimensional (2D) or three-dimensional (3D) nanostructures via complementary pairing with short staple DNA strands [160]. M13 scaffold plus a 40 × excess of staples achieved isothermal self-assembly at 25 °C, creating structures like triangles, rectangles and smileys (Fig. 11a) [160]. Hydrogen bonds and van der Waals forces between base pairs determine the specificity and stability of self-assembled architectures, enabling programmable control of nanoscale precision (Fig. 11b). Resulting DNA nanostructures, including nanotubes, hexagonal lattices, six-helix bundles, 3D crystalline arrays, and polyhedral structures [161], exhibit programmable geometries and functionalities, serving as platforms for supramolecular engineering (Fig. 11c).

In drug delivery, the programmability of DNA nanostructures enables creation of smart nanodevices with tailored functionalities. Consequently, these nanostructures can encapsulate therapeutic payloads (such as thrombin, antigens, and siRNA) within cavities or on surfaces through various binding methods [162]. For example, tubular DNA nanorobots protect thrombin until nucleoli-targeting aptamers trigger site-specific release in tumors, inducing localized thrombosis and tumor regression [163]. Similarly, DNA origami vaccines co-loaded with antigens and adjuvants use pH-responsive locks to expose immunostimulatory components in lysosomes, enhancing T-cell activation [164]. Moreover, the spatial arrangement of ligands (such as aptamers and peptides) on icosahedral DNA frameworks further optimizes cell-targeting efficiency and internalization pathways [165]. This programmable approach enables development of complex delivery systems for therapeutic applications. In 2020, a bacterial-targeted platform, using DNA origami with aptamer technology, modified by lysozyme to achieve specific bacterial growth inhibition (Fig. 11d) [166]. This offers precise targeting and simultaneous delivery of multiple antibacterial agents, providing a tool for addressing antibiotic resistance.

Notably, recent advancements highlight the simplicity and versatility of tetrahedral framework nucleic acids (TFNAs) in drug delivery. TFNAs represent a distinct class of DNA nanostructures bridging DNA origami and supramolecular self-assembly principles. Unlike traditional DNA origami that folds long single-stranded DNA with staple strands for complex 2D/3D shapes, TFNAs form through self-assembly of four oligonucleotides into a tetrahedral architecture via one-pot annealing [167]. The strands form a framework through three-way junctions, creating a three-dimensional scaffold with customizable vertices and edges for precise modifications [168]. This design provides TFNAs with structural stability, programmable functionality, and biocompatibility. Their stability stems from tetrahedral architecture rigidity and nuclease resistance. In monovalent ion environments (Na^+^, K^+^, Li^+^), these frameworks show nuclease resistance 10-fold greater than in divalent conditions (Mg^2+^, Ca^2+^, Ba^2+^) (Fig. 11e) [169].

TFNAs' hollow cavity and modifiable surfaces enable various drug-loading strategies (Fig. 11e): Ⅰ) Covalent conjugation. Carbon brominated camptothecin reacts with PS-modified DNA to form drug-grafted DNA with responsive disulfide bonds [170]. In addition, Bone Morphogenetic Protein-2 binding peptide was modified on TFNA via click chemistry of azido-alkyne addition [19]. Ⅱ) Noncovalent complexes. Therapeutic molecules form complexes with TFNAs through noncovalent interactions. For example, the cationic antimicrobial GL13K peptide adsorbs onto TFNAs via electrostatic interaction [171]. Ⅲ) Groove-binding and embedded-binding. Lipoic acid [172], puerarin [173], doxorubicin [174], and typhaneoside [175] embed into TFNA groove regions. Ⅳ) Encapsulation by physical diffusion. Quercetin enters TFNA's internal cavity through physical diffusion [176].

Due to their programmability, TFNAs have been widely applied in drug delivery. In size-based nucleic acid sequence design, chains of varying lengths pair complementarily to form TFNAs with tunable particle sizes, which are crucial factors in transdermal delivery systems. Wiraja et al. synthesized three tetrahedrons: TH21 (21 base pairs), TH37 (37 base pairs), and TH337 (337 base pairs), with average diameters of 17, 44, and 187 nm, respectively (Fig. 11f1) [174]. TH21 and TH37 achieved transdermal penetration to 350 μm depth, whereas the larger TH337 primarily accumulated in the epidermis (50–75 μm from the surface) (Fig. 11f2). This size-dependent penetration behavior aligns with the transdermal mechanism of tetrahedral FNAs, which is dominated by size-dependent physical penetration. Approximately 75 %–80 % of tetrahedral FNAs penetrate into the dermal layer via the appendageal pathway, while the intercellular pathway accounts for a smaller proportion (20 %–25 %). This is attributed to their non-deformable rigid structure, which hinders their passage through narrow intercellular gaps; meanwhile, this rigidity also endows FNAs with excellent structural stability, rendering them efficient local transdermal drug carriers. Consequently, TH21 loaded with DOX exhibited higher transdermal efficiency than free DOX, liposomes, and polymer nanoparticles, accompanied by lower toxicity (Fig. 11f3 and 11f4). In sequence-based programmable design, covalent therapeutic conjugation, environment-responsive modification, and targeted delivery have become the focus points of research. Li et al. linked RNase A to thiol-modified DNA strand S1 via 4-nitrophenyl 2-(2-pyridyldithio) ethyl carbonate, while PTK7 receptor-targeting sgc8 aptamer in strands S2/S3 provided targeting capability. High GSH in cancer cells triggered bond cleavage, releasing RNase A [177]. TFNAs’ editable topology and functional modification make them promising SAMs for addressing bioavailability, targeted delivery, and controlled release challenges.

### 4.4 Summary of next-generation SAMs

The preceding text focuses on the core proposition of programmability of next-generation supramolecular SAMs and systematically analyzes their core characteristics and design strategies from three levels: environmentally responsive driving forces, component unit design, and precise rule-based encoding. This section will conduct a systematic inductive and comparative analysis of the emerging SAMs discussed previously from five key dimensions: building blocks, dominant driving force, typical structure, key advantages, and critical limitations. The key elements are summarized in Table 2 to visually present their commonalities and unique characteristics, providing a reference for subsequent research or technology selection.

**Table 2:** Comparative analysis of next-generation supramolecular SAMs.

SAMs	Building Blocks	Driving Force	Typical Structure	Key Advantages	Critical Limitations	Ref.
Lipid Nanoparticles	Phospholipids	Hydrophobic Interaction	Vesicles	Nucleic acid protection/delivery; mature preparation process; feasible engineering application	Immunogenicity/safety risks; insufficient targeting ability; poor storage stability	[178]
Natural Free-Carrier Assembled Materials	Natural herbal components	π-π Stacking, Hydrogen Bond	Nanocrystals, Supramolecular Gels	Good biocompatibility/biodegradability; high drug-loading capacity; natural origin; simple synthesis	Complex self-assembly mechanism; poor process controllability; limited in vivo stability; long R&D cycle	[[179], [180], [181]]
Amphiphilic Block Copolymer micelles	Polymer	Hydrophobic Interaction	Micelles	Enhanced drug solubility; tunable physicochemical properties; programmable functions	Immunogenicity/safety risks; dilution instability; protein adsorption; drug leakage	[[182], [183], [184]]
Ionic Liquids and Deep Eutectic Solvents	Organic cations and anions (ILs), Lewis or Brønsted acids and bases (DESs)	Electrostatic Interaction (ILs), Hydrogen Bond (DESs)	Nanoparticles	Enhanced drug solubility and bioavailability; auxiliary transdermal delivery; simple synthesis; high thermal stability	Potential toxicity; limited physicochemical properties; high cost; unknown biodegradability	[185,186]
Self-Assembled Peptides	Amino acid sequences	Hydrophobic Interaction, Hydrogen Bond	Nanofibers, Nanotubes, Hydrogels	Excellent biocompatibility/safety; biodegradable; highly programmable	Large-scale production/high cost; poor stability; easy degradation in vivo; uncontrollable drug release	[187]
Polysaccharide Assembled Materials	Natural Polysaccharides	Hydrogen Bond	Inclusion Complexes, Nanogels	Excellent biocompatibility/safety; low cost; wide source availability; tunable structure/function	Poor production batch stability	[188]
Metal-Organic Framework	Metal ions/clusters and Aromatic polyacid or polyamine	Metal-Ligand Coordination Bond	Rigid porous frameworks	Extremely high porosity/specific surface area; high drug-loading capacity; tunable and easily modifiable pore structure	Potential toxicity of metal ions; difficulty in synthesis/scale-up; easy decomposition in physiological environment	[189,190]
Metal-Phenolic Networks	Metal ions and Polyphenols	Metal-Ligand Coordination Bond	Nano-fiber network or membrane	Dynamically tunable; high bioadhesion/encapsulation efficiency	Potential toxicity of metal ions; difficulty in scale-up; poor in vivo stability	[191]
DNA Origami	DNA strands	Hydrogen Bond	Tetrahedrons, Origami shapes (2D/3D)	Excellent biocompatibility/safety; precisely controllable shape/size; high biofunctionalization/programmability	High cost in large-scale production; high degradation risk; short in vivo half-life	[192]

## 5 Programmable design and functional advantages of supramolecular SAMs

The monomers of SAMs are not connected by covalent bonds; instead, they form ordered aggregates through non-covalent interactions, thereby exhibiting unique properties and functions that individual monomer molecules lack. Inspired by biological systems, supramolecular self-assembled materials provide an elaborate bottom-up construction strategy for the creation of novel materials. In this section, we clarify the design concept and direction of programmable design for next-generation supramolecular self-assembled materials, and point out that such materials exhibit two categories of unique structural advantages, which will be elaborated in detail in the subsequent content.

### 5.1 Programmable design for functional integration of SAMs

**Fig. 12:**
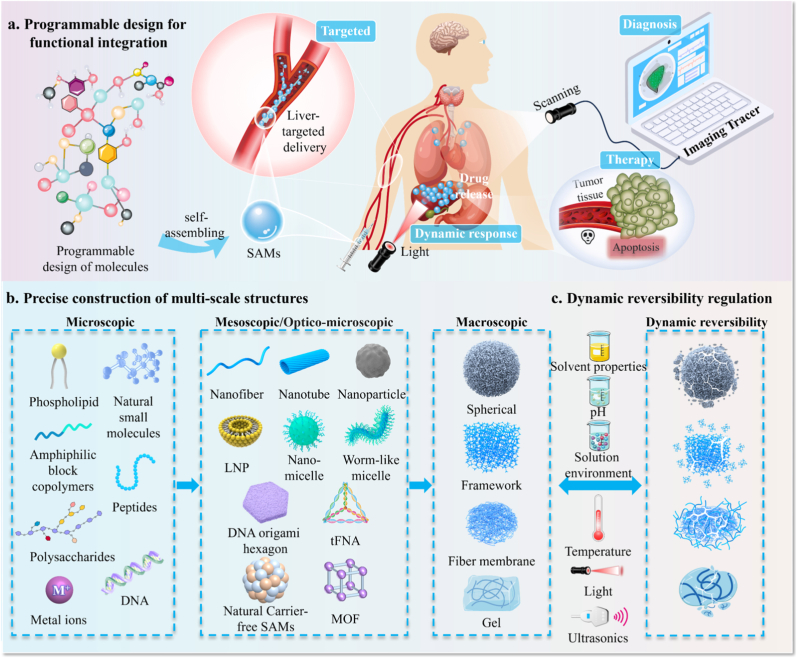
Functional advantages of supramolecular SAMs. **a)** The programmable design and functional integration of self-assembly systems. **b)** Precise construction of multiscale structures: from the microscopic molecular structure, to the mesoscopic/optico-microscopic nano structural units, and finally to the macroscopic aggregates. **c)** Dynamic reversibility regulation of self-assembly system: environmental impact on dynamic assembly process.

The next-generation of supramolecular SAMs features programmability, multifunctionality, and intelligent responsiveness. The multifunctionality and intelligent response characteristics of SAMs originate from their programmable molecular design concept. Through the precise regulation of intermolecular interactions, these materials can achieve dynamic tunability and environmental adaptability, thereby enhancing the stability of the assembly system [193]. Despite the significant differences in their building blocks (including DNA, polypeptides, and polymers), the core logic for realizing molecular programming is consistent, enabling the fabrication of multifunctionally integrated supramolecular self-assembled materials. As illustrated in Fig. 12a, during the programmable design of materials at the molecular level, their structural modules (e.g., DNA scaffold strands, polypeptide backbones) mainly serve to provide morphological support and structural stability; functional modules (e.g., RGD peptides, nucleic acid aptamers, polyethylene glycol chains) are introduced via surface modification or co-assembly to endow the materials with active targeting or long-circulation capability; response modules (e.g., pH-sensitive hydrazone bonds, ROS-sensitive thioacetal bonds, enzymatic cleavage sites) can act as intelligent switches to precisely regulate the timing of drug release, thus achieving efficient therapeutic efficacy. In addition, as carriers, these materials can load imaging markers (e.g., fluorescent probes, radioisotopes) and combine with clinical imaging techniques to realize accurate localization and early diagnosis of lesions. Taking "molecular programmable design" as the core, this technical route runs through the entire process of medical function integration, providing an integrated technical solution for the field of precision medicine.

This design enables multifunctional integration, advancing supramolecular materials toward intelligence and precision. For example, based on the high programmability of DNA sequences, Wang et al. developed a modular tumor treatment system integrating DNA origami, disulfide bond gating, targeted delivery (TAT peptide), and combined therapy (siRNA + DOX) [194]. Peptide molecules, with editable amino acid sequences, form another important class of self-assembling units. Wang et al. modified RADA16-I by adding Tβ4 therapeutic agents and RGDs cell adhesion ligands. The resulting three-dimensional hydrogel network promoted epicardium-derived cell adhesion, with Tβ4 continuously release for 28 days activating epicardium and enhancing infarcted myocardium repair [195]. In synthetic polymers, block copolymers show strong self-organizing capabilities. Liu et al. developed a self-assembled photonic microsensor using bottle-brush block copolymers (BBCP) of polystyrene (PS) and polyethylene oxide (PEO) with grafted TPE groups, achieving ppb-level detection of nitro phenolic compounds through structural color and fluorescence signals [196].

In conclusion, supramolecular SAMs exhibit great potential in the field of drug delivery. By leveraging their strengths, researchers must also identify their limitations and optimize the design based on a profound understanding of their characteristics.

### 5.2 Precise construction of multiscale structures (Fig. 12b)

#### 5.2.1 Precise control of nanoscale structures

During self-assembly of amphiphilic polymer molecules, non-covalent interactions introduce an additional layer of kinetic control, ensuring precise control over the aggregation and growth into micelles [197]. The size, aggregation number, and cloud point of micelles can be controlled by the composition and chain length of the copolymer's alkyl side chains. Among these parameters, micelle size (Rh) is dependent on the proportion of hydrophobic monomers and alkyl side chain length rather than the main chain length, exhibiting a gradual increase with the elevated proportion of hydrophobic monomers and elongated alkyl side chains: Rh (Cn) = 3.7 nm (C4), 4.6 nm (C8), 6.4 nm (C12), and 14 nm (C18) [198]. Higher proportions of hydrophobic blocks in the copolymer leads to an expansion of the micellar core volume, transforming morphology from low curvature (spherical) to high curvature (vesicular) [199]. DNA origami technology has progressed from Holliday junction structures to two-dimensional planes and complex three-dimensional structures. Through base complementarity, this technology enables precise regulation of nanoscale features, allowing precise design of DNA nanotube/bandwidth and diameter through bottom-up self-assembly, with a width adjustable range of 11–70 nm, length scalable to the micrometer level, and support for biomolecular patterning with sub-10 nm precision [200]. He et al. reviewed two-dimensional and three-dimensional structures self-assembled using DNA origami technology, showing that both simple two-dimensional structures (triangles, stars, smiling faces, hexagonal tiles) and complex three-dimensional structures (tetrahedrons, hexahedrons, octahedrons, icosahedrons) can be achieved through customized assembly [32,[201], [202], [203]].

#### 5.2.2 Cross-scale structure control

While achieving precise control of nanoscale structures, this process can break through scale limitations to form macroscopic assemblies. Peptide molecules form β-sheet/α-helix secondary structures through spontaneous means, driving their self-assembly into 1D, 2D, and 3D nanostructures [204]. The S-CP peptides developed by Liu et al. self-assemble into highly ordered cubic frameworks via chirality-induced helix structure, exposing the peptide chain's main chain and side chains to control assembly direction through site-specific mutations to achieve lateral stacking [205]. Peptides dominated by β-sheets typically self-assemble into nanofiber networks to construct hydrogel scaffolds. When containing many hydrophilic amino acid residues, it can self-assemble into ordered nanostructures like nanofibers, nanotubes, or vesicles [206]. Garcia et al. synthesized Pro-Phe-Phe, combining β-breaker motif and β-structure-associated diphenylalanine motif. XRD and molecular dynamics simulations showed the peptide can undergo long-range (gel fiber stacking), medium-range (nanobelt stacking), or short-range (dimer/trimer nanoparticles) assembly, with assembly behavior related to amino acid chirality [207].

### 5.3 Dynamic reversibility regulation of self-assembly (Fig. 12c)

Supramolecular self-assembly is a spontaneous process driven by thermodynamics and kinetics. The intermolecular non-covalent interactions can be precisely regulated by manipulating external environmental parameters (pH, ionic strength, temperature, *etc.*), which influence the dynamic reversibility and response characteristics of the assembly structure by altering the molecule-solvent interactions. Weerakkody et al. demonstrated that adjusting the water content and pH of surface-active peptide CG7-NH_2_ regulates the equilibrium among monomers, nanofibers, and nanofiber-spherical aggregates [208]. The pH-responsive self-assembling peptide system developed by Schneider's team shows that the polypeptide exists in an unstructured solubilized state under acidic conditions (pH 5.5). As the pH increases to 9.0, the polypeptide undergoes intramolecular folding to form an amphiphilic β-hairpin structure, which further self-assembles into a hydrogel. Conversely, readjusting the pH to 6.0 reverses the folding behavior and induces gel dissolution, verifying the high reversibility of this transformation. Notably, this hydrogel possesses superior self-healing performance: it displays shear-thinning behavior under shear stress and can recover 80 % of its initial modulus within 30 min after the removal of shear stress [209]. These responsive hydrogel materials have been widely applied in biomedical and engineering fields. The “on–off” state transitions of their assembly structure can be controlled by external stimuli (e.g., electricity, light, heat, or pH), modifying the material's properties and enabling reversible sol-gel transitions. Particularly, smart materials capable of gel-sol transitions at physiological temperatures offer ideas for next-generation biomaterials [207,210]. Most self-assembly systems exhibit dynamic reversibility, as exemplified by two typical cases: DNA origami structures that switch between “+” and “X” configurations in response to pH variation [211], and PDMS materials containing hydrogen bonds and disulfide bonds that display an ultrahigh elongation ratio of 14,000 % along with room-temperature self-healing capability [212]. Notably, the PDMS material can still restore its mechanical strength even after enduring multiple cycles of mechanical stress, which corroborates the high robustness of its assembly-disassembly cycle. By maintaining the structural integrity of the gel matrix, this rapid sol-gel transition and modulus recovery mechanism can efficiently suppress the burst release of drugs under shear stress conditions (e.g., intravascular blood flow or injection procedures), thereby ensuring the long-term retention and sustained release of payloads.

## 6 Application of supramolecular SAMs drug delivery systems in disease treatment

**Fig. 13:**
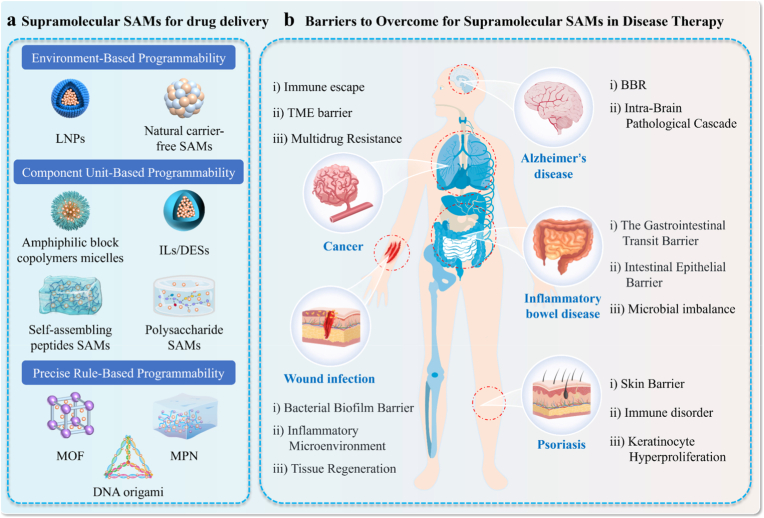
SAMs in disease treatment. **a)** The next-generation SAMs loaded with drugs. **b)** Barriers to Overcome for Supramolecular SAMs in Disease Therapy.

Supramolecular SAMs, designed through intermolecular weak interactions, exhibit significant multifunctionality. Upon administration, supramolecular self-assembled drug delivery systems face competition between physiological ions and organic ligands, causing disassembly and drug release. With multidisciplinary integration, more SAMs have been developed (Fig. 13a). SAM-based DDS show immense potential in treating various diseases, attracting research interest. This chapter summarizes the obstacles that need to be overcome in the treatment of various diseases (Fig. 13b) and elaborates on the application and progress of supramolecular SAMs in drug delivery for different diseases.

### 6.1 Cancer therapy

Cancer remains a formidable global health challenge, characterized by uncontrolled cell proliferation, invasiveness, and metastatic potential. It imposes heavy physical and psychological burdens on patients, financial strain on families, and substantial pressure on healthcare systems. While conventional treatments such as surgery, chemotherapy, and radiotherapy are mainstays of clinical oncology, their efficacy is often limited by pathological barriers that shield tumors and impede efficient drug delivery. The tumor microenvironment (TME) mediates immune suppression and facilitates immune escape of tumor cells. Its heterogeneity and complexity not only form substantial physical barriers that impair the enhanced permeability and retention (EPR) effect, but also trigger P-glycoprotein overexpression via inter-tumoral heterogeneity, thereby exacerbating multidrug resistance (MDR) through drug efflux [213,214]. The development of advanced therapeutic strategies hinges on overcoming these multi-layered defense mechanisms. Supramolecular SAMs provide a versatile platform to systematically address these barriers. Table 3 summarizes the applications of SAMs as DDSs in tackling key obstacles to tumor therapy. Their programmable properties enable the design of DDSs capable of navigating systemic circulation, accumulating at tumor sites, achieving deep tissue penetration, releasing therapeutic payloads in response to specific TME triggers, and concurrently modulating host immune responses.

**Table 3:** SAMs for cancer treatment.

SAMs	Building Blocks	Morphology	Drugs	Therapeutic Mechanisms	Ref.
Peptide SAMs	KFM peptide	Hydrogel	KFM, Mitoxantrone, Metformin	Gel@MTX/MET: KFM hexapeptide core, self-assembles to ROS-responsive hydrogel; dual breakthrough (TME immunosuppression & tumor immune escape barrier); promotes CD8^+^ T/NK cell infiltration; inhibits tumor growth	[20]
MOF	Cu^+^, BBTZ, Mo_8_O_26,_ TAB^+^	Nano-crystal (CCUT-1)	DOX, CCUT-1	CCUT-1@Gel efficiently loads chemotherapeutic drugs via the porous MOF structure, while the dual-layer hydrogel enables targeted sustained release and low-toxicity delivery.	[22]
LNP	Various lipids	Spherical LNPs	5-Fluorouracil, Chimeric nanobody	Chimeric nanobody-modified LNPs leverage small size and HER2-targeting nanobodies to overcome tumor microenvironment barriers and low Enhanced Permeability and Retention effect, achieving precise tumor accumulation.	[53]
Natural carrier-free SAMs	Celastrol (Cel), Erianin (Eri)	Spherical NPs	Cel, Eri	Self-assembly NPs (CENs) achieves precise tumor enrichment via small spherical size and EPR effect, enhances bioavailability, reduces toxicity, and synergistically inhibits breast cancer growth.	[85]
Saccharide SAMs	Hyaluronic acid, Chloroquine (CQ)	Nanogel	Cisplatin, CQ	Nanogels with spherical small particle size + CD44 targeting, achieve precise tumor accumulation, address low EPR effect and drug delivery obstruction by physical barriers, improve the efficiency of drug nuclear entry.	[134]
MPN	Fe^2+^, Rosmarinic acid (RA)	Spherical NPs	Fe-RA	Fe-RA nanocomposite targets tumor tissues via the EPR effect, with its porous structure and coordination properties endowing peroxidase-like activity and glutathione-depleting capacity.	[154]
DNA origami	dsDNA, Cationic conjugated polymers	Tetrahedral NPs	AS 1411, DOX, G3139	DNA framework-CCP hybrid materials efficiently load dual types of drugs via synergistically formed high-affinity drug pockets, and enhance cellular uptake and circulatory stability through near-neutral charge and stable structure.	[216]
Peptide SAMs	Ac-(RADA)_4_-CONH_2_	Nanofiber hydrogel	CUR, DOX	The hydrogel serves as a local depot for Fickian drug release; DOX intercalates DNA for cytotoxicity, CUR modulates cell cycle/apoptosis, enhancing drug uptake and tumor cell apoptosis.	[217]
Amphiphilic block copolymer materials	C18-sgc8	Nanomicelles	Ce6	C18-aptamer nanomicelles via tandem C18 chains stabilization plus aptamer targeting achieve precise tumor accumulation, improve low EPR effect, enhance hydrophobic drug internalization and antitumor efficacy.	[218]
Natural carrier-free SAMs	Genistein (Gen), Chlorin e6 (Ce6)	Spherical NPs	Gen, Ce6	GC NPs and CEN with spherical small size enable precise tumor enrichment, enhance bioavailability, reduce toxicity, and synergistically inhibit tumors via structural advantages.	[219]
Natural carrier-free SAMs	Oleanolic Acid(OA)	Nanomicelles	OA	OA spherical nanomicells with appropriate dimensions and stable structure, recruit immune cells for infiltration, enhance immune response at tumor site, alleviate immunosuppressive state of TME, and reduce immune escape of tumor cells.	[220]
DNA origami	Four ssDNA	Six-helix bundle	Maytansine	DNA framework carriers use programmable asymmetric hydrophobic drug shapes to overcome TME barrier issues and low ERP effect, delivering DM1 across membranes via dual pathway.	[221]
DNA origami	Four ssDNA	Tetrahedral NPs	siRNA, AS 1411	tFNAs-AS1411-siBraf overcomes siRNA delivery barriers via tetrahedral small size and aptamer targeting, efficiently targets and internalizes into melanoma cells for precise gene therapy.	[222]

TME-responsive drug release and therapy rely on the unique chemical signatures of the TME with low pH and high ROS. These signatures are widely exploited as triggers for intelligent drug delivery. SAMs can be engineered with acid-labile bonds that hydrolyze in the acidic TME, or with ROS-cleavable linkers (e.g., thioketals) that break under oxidative stress. This design ensures preferential release of therapeutic payloads at the tumor site, maximizing local concentration and minimizing systemic exposure. Additionally, some SAMs can leverage TME components for therapeutic effects via chemodynamic therapy (CDT) [22]. In CDT, materials catalyze the conversion of endogenous H_2_O_2_ into highly toxic hydroxyl radicals (·OH) to induce cancer cell death [215]. SAMs also serve as new tools for cancer immunotherapy. They can be designed to deliver immune checkpoint inhibitors or small molecules that interfere with immunosuppressive pathways. More innovatively, peptide-based SAMs can directly interact with and degrade checkpoint proteins (e.g., PD-L1), which effectively unmasks tumors for immune attack [20]. Meanwhile, co-delivery of chemotherapeutics inducing immunogenic cell death and immunomodulators via SAMs enables a potent synergistic anti-tumor immune response. To address delivery barriers, SAMs are often functionalized with targeting ligands (e.g., antibodies, aptamers) that bind to overexpressed receptors on cancer cells, a strategy known as active targeting. This complements passive accumulation via the EPR effect [53]. For overcoming MDR, SAMs can co-encapsulate chemotherapeutic agents with MDR inhibitors or second-line-drugs with distinct mechanisms of action. This synergistic co-delivery ensures simultaneous arrival of both agents at cancer cells to overwhelm resistance mechanisms. DNA nanostructures with precise addressability are particularly suitable for constructing complex multi-agent delivery vehicles [85,216].

### 6.2 Alzheimer's Disease therapy

Cardiovascular, cerebrovascular, and neurological diseases pose a severe threat to human health. Among these disorders, Alzheimer's disease (AD) is associated with high rates of disability and mortality. As the most prevalent form of dementia, AD is a devastating neurodegenerative disorder characterized by progressive cognitive decline. The therapeutic landscape for AD remains highly challenging, primarily owing to the formidable biological barriers protecting the central nervous system (CNS) and the disease's intricate, multifactorial pathogenesis. Effective AD treatment is further impeded by critical drug delivery hurdles and a complex network of interconnected pathological processes within the brain. The blood-brain barrier (BBB) is the CNS's primary selective semipermeable barrier, consisting of endothelial cells reinforced by tight junctions, enzymatic activity, and active efflux transporters (e.g., P-glycoprotein). It blocks small-molecule and large-molecule biologics from non-selectively entering the CNS extracellular fluid, thus becoming the major obstacle to CNS therapeutic development [223]. Additionally, any drug crossing the BBB must target the core pathological cascade of AD, including extracellular amyloid-beta (Aβ) plaque aggregation, intracellular neurofibrillary tangles (NFTs) caused by hyperphosphorylated tau, and subsequent chronic neuroinflammation, synaptic dysfunction, oxidative stress, and neuronal death. This emphasizes that effective therapies must achieve both BBB penetration and targeted modulation of these pathological processes [224,225]. SAMs are being engineered with sophisticated features to address both the delivery and efficacy challenges in AD therapy. Table 4 summarizes the applications of SAMs as DDSs in tackling key obstacles to AD therapy. These systems are designed to carry therapeutic agents across the BBB, delaying neurodegenerative processes by protecting neuronal function, modulating neurotransmitter balance, and suppressing inflammatory responses.

**Table 4:** SAMs for Alzheimer's disease (AD) treatment.

SAMs	Building Blocks	Morphology	Drugs	Therapeutic Mechanisms	Ref.
DNA origami	Four ssDNA	Nanoflowers	miR-124, RVG29 peptide, Rutin	Rutin@DF-miR-124/RVG29, 200-nm DNA nanoflowers. RVG29 receptor-mediated penetrates BBB, targets neurons; co-loads miR-124/rutin, pH-responsive release. Synergistically inhibit BACE1/APP to reduce Aβ, rutin antioxidant scavenge ROS, improve cognition.	[226]
Natural carrier-free SAMs	Donepezil, Simvastatin	Spherical NPs	Donepezil, Simvastatin	NanoDS, donepezil-simvastatin self-assembled spherical NPs (70 nm), intranasally bypasses BBR for efficient brain delivery, synergistically improve synaptic plasticity, clear Aβ, anti-inflammatory/antioxidant, address AD's symptoms/etiology.	[228]
MPN	Mn^2+^, IR780@TA NP	Spherical NPs	TA, TPL peptide	IR780-Mn@TA-TPL, spherical NPs (116 nm) with TPL modification, achieving BBB penetration via TPL targeting to reduce oxidative stress and tau pathology in AD, enhancing cognition.	[229]
Peptide SAMs	KLVFF, 5-(4-carboxyphenyl)-10,15,20-triphenylporphyrin	Spherical NPs	Self-assembled complex	PKNPs penetrate the BBR via the photothermal effect of their spherical nanostructure, disassemble into an amorphous form upon encountering Aβ, activating photodynamic activity to selectively oxidize and inhibit Aβ aggregation.	[230]
Peptide SAMs	FEFEFEFE and FKFKFKFKGGRGDSP	Nanofiber	CRISPR/dCas9	PCL nanofibers layer-by-layer modified with self-assembling peptides form bioactive scaffolds, load/sustainably release CRISPR/dCas9 complexes via hydrophobic/electrostatic interactions, SAP^+^-RGD enhances cell adhesion/proliferation.	[232]
LNPs	Various lipids	Spherical LNPs	Berberine and siRNA(siBACE1)	BE-LNPs, berberine-inspired 70 nm spherical LNPs, with stable nucleic acid loading and endosomal escape, penetrate blood-brain barrier via dopamine D3 receptor-mediated brain targeting, deliver nucleic acids, suppress markers, improve cognition, overcome barriers.	[233]
LNPs	Various lipids	Spherical LNPs	Cardiolipin, CUR	RCLs@CNPs, 70 nm spherical LNPs, intranasally bypass blood-brain barrier, release CNPs/cardiolipin in Alzheimer's disease oxidative microenvironment. Inhibit Aβ aggregation, regulate TLR4/NF-κB, synergistic Aβ clearance plus anti-inflammation.	[234]
DNA origami	Four ss DNA	Tetrahedral NPs	Nucleic acid framework	TDNs, 12-nm tetrahedral DNA nanostructures, partially penetrate BBB, inhibit mitochondrial apoptosis pathway (downregulate Bax/caspase-3, upregulate Bcl-2) to reduce Aβ deposition, improve learning memory in Alzheimer's disease models.	[235]
DNA origami	Four ss DNA	Tetrahedral NPs	Nucleic acid framework	TFNAs bypass BBB by intranasal delivery and target hippocampal neurons, downregulate Atf3 upregulate Rrm2 Furin inhibit Aβ ferroptosis improve cognition.	[236]
MOF	Fe^3+^, trimesic acid (TMA)	Spherical NPs	Ceria, siSOX9, Retinoic acid	CeRMS, H_2_O_2_-responsive release drugs to promote neuronal differentiation and scavenge ROS. NSC transplant to crosse BBB and shown neuroprotection, improves cognition in AD models.	[237]
MOF	Mn^2+^, 2,5-dihydroxyterephthalic acid(H_4_DOBDC)	Hexagonal rod-shaped NPs	Dihydroquercetin, Resveratrol	DR@MOF, hexagonal rod-like NPs; sequential release drug via adsorption energy differences, Oral reduces brain oxidative stress Aβ deposition, ameliorates cognition in Alzheimer's models.	[238]
MOF	Ce^3+^, Zr^4+^, 1.4-phthalic acid (H_2_BDC)	Spherical NPs	Lactoferrin, CUR	Ce/Zr-MOF@Cur-Lf, spherical MOF-based NPs loaded with curcumin and modified with lactoferrin(Lf). It penetrates the BBB via Lf receptor-mediated targeting, scavenges ROS, reduces Aβ deposition and neuroinflammation in the brain, achieving multi-target therapy.	[239]
MPN	Fe^2+^/Fe^3+^, TPP-quercetin (TPP-QC)	Spherical NPs	TPP, QC	TQCN, TPP-modified spherical NP, bypasses the BBB via intranasal delivery, targets mitochondria to chelate iron ions to self-assembles in situ, and activates the Nrf2 pathway to restore iron homeostasis and antioxidant defenses, ameliorating AD pathology.	[240]

Strategies for BBB penetration using SAMs rely on multiple mechanisms. First is receptor-mediated transcytosis. SAM-based nanoparticles are decorated with specific ligands, such as the peptide RVG29 or antibodies against the transferrin receptor [226]. These ligands bind to receptors on the BBB endothelial cells, inducing the cells to transport the entire nanoparticle into the brain parenchyma. Second is non-invasive intranasal delivery. This route bypasses the BBB by enabling direct drug transport from the nasal cavity to the brain via olfactory and trigeminal nerves [227,228]. It offers a promising alternative for CNS drug delivery. The third strategy is physicochemical modification. This includes engineering small, lipophilic nanoparticles or applying external stimuli like focused ultrasound or light [229]. These measures transiently enhance the BBB permeability. Upon reaching the brain parenchyma, SAMs can be programmed for targeted therapeutic effects against core AD pathologies. They can incorporate peptides homologous to the Aβ core region (e.g., KLVFF) as "beta-sheet breakers" to bind and disrupt Aβ aggregates [230]. SAMs can also deliver kinase inhibitors to reduce tau hyperphosphorylation or agents to inhibit tau aggregation. Additionally, SAMs can be composed of or loaded with antioxidant molecules, such as curcumin or tannic acid. These molecules neutralize excessive reactive oxygen species in the AD brain, protecting neurons from oxidative damage and alleviating neuroinflammation [231].

### 6.3 Inflammatory bowel disease therapy

Gastrointestinal diseases significantly impact human health. Inflammatory bowel disease (IBD), encompassing Crohn's disease and ulcerative colitis, is a chronic inflammatory condition of the gastrointestinal (GI) tract. Therapy aims to reduce inflammation and promote mucosal healing, but conventional treatments are often plagued by systemic side effects and a lack of specificity for the diseased tissue. Effective IBD treatment requires navigating the harsh GI environment to deliver drugs specifically to the inflamed intestinal segments while also addressing the underlying pathology. Oral drug delivery is the preferred route for treating IBD, but it faces multiple challenges. Drugs must withstand the strongly acidic gastric environment and resist enzymatic degradation in the small intestine. In addition, rapid gastrointestinal transit and the intestinal wall's protective mucosal layer prevent drugs from reaching and remaining at the inflamed colonic sites where they are most needed [241]. The core pathological feature of IBD is intestinal epithelial barrier dysfunction. It is marked by the relaxation of interepithelial tight junctions, which leads to increased intestinal permeability, known as leaky gut. This permeability allows antigens and bacteria in the intestinal lumen to invade deep tissues and exacerbate the inflammatory vicious cycle [242]. Thus, repairing this epithelial barrier is a key therapeutic goal. Meanwhile, IBD is linked to gut dysbiosis, an imbalance in the composition and function of gut microbiota. Beneficial bacteria decline in number, while pathogenic or pro-inflammatory bacteria overproliferate. This dysbiosis, together with disordered mucosal immune responses, jointly drives chronic inflammation [243]. Therefore, treatment should balance inflammation inhibition and microbial homeostasis restoration. SAMs are ideally suited to overcome these challenges by providing protective encapsulation, targeted delivery, and modulation of the gut microenvironment. Table 5 summarizes the applications of SAMs as DDSs in tackling key obstacles to IBD therapy. These materials restore intestinal function by inhibiting inflammatory responses, repairing the mucosal barrier, modulating gut microbiota, and promoting tissue regeneration.

**Table 5:** SAMs for inflammatory bowel disease (IBD) treatment.

SAMs	Building Blocks	Morphology	Drugs	Therapeutic Mechanisms	Ref.
Natural carrier-free SAMs	Berberine (BBR), Glycyrrhizic acid (GA)	Nanofiber hydrogels	GA, BBR	GA-BBR hydrogel, a self-assembled nanofiber, shows pH-responsive release and mucoadhesion. Rectal delivery bypasses GI barriers, targets colon, suppresses TNF-α/IL-6, repairs epithelial integrity, and modulates immunity without altering microbiota, demonstrating efficacy in UC therapy.	[75]
Amphiphilic block copolymer materials	Methoxy poly-(ethylene glycol) hexyl substituted poly-(lactic acid) (mPEGhexPLA)	Spherical NPs	Cyclosporine A (CsA)	mPEGhexPLA (50 nm spheres) deliver CsA via rectal administration to UC lesions. Their small size and neutral charge enable penetration of the impaired intestinal barrier, releasing CsA locally to inhibit inflammation without altering gut microbiota, addressing gastrointestinal and immune barriers.	[244]
Polysaccharide SAMs	Chitosan (CS), Fucoidan (FU)	Spherical NPs	Rhein	RH-F/C-NPs are CS/FU-based spherical NPs with pH/ROS sensitivity. Oral delivery targets colon release of RH, inhibiting TLR4/NF-κB inflammation, activating Nrf2/HO-1 antioxidant pathway, repairing intestinal barrier, and regulating microbiota to address GI barriers, epithelial dysfunction, and immune/microbial dysregulation in UC.	[245]
Natural carrier-free SAMs	Aromatic dithiol (BDT), Tannic acid (TA)	Spherical NPs	TA, Dexamethasone sodium phosphate	EcN@SA-pBDT-TA, a polyphenol-modified probiotic, enables oral colon targeting with GI resistance. It scavenges ROS, suppresses TNF-α/IL-6, upregulates tight junction proteins (e.g., ZO-1), and modulates microbiota (e.g., Akkermansia), addressing GI barriers, epithelial dysfunction, and immune dysregulation in IBD.	[246]
LNPs	Various lipids	Spherical LNPs	IL-22-mRNA	Spherical LNPs (∼200 nm) by Oral delivery enables colon-targeted IL-22 mRNA expression, enhancing epithelial barrier integrity, suppressing inflammation, and accelerating UC recovery without gut microbiota alteration.	[247]
LNPs	Various lipids	Spherical LNPs	IL-10-mRNA	Spherical LNPs with 30 % DSPC, target inflamed colons via IV delivery, express IL-10 mRNA to suppress pro-inflammatory cytokines (e.g., TNF-α, IL-6), restore intestinal barrier integrity, and modulate immunity without altering gut microbiota, demonstrating efficacy in IBD models.	[248]
Amphiphilic block copolymer materials	PEG-1,4-dihydrophenathrolin-4-one-3-carboxylic acid (DPCA)	Hydrogel	DPCA	PEG-DPCA hydrogel delivers DPCA subcutaneously, stabilizing HIF-1α to upregulate barrier genes (MUC2, TFF3) and induce EMT for epithelial repair. It suppresses TNF-α/IL-6 and promotes Treg-mediated immunomodulation without gut microbiota alteration, effectively treating experimental colitis.	[249]
Polysaccharide SAMs	Hyaluronic acid (HA), Histidine (His), Luteolin (LUT)	Hydrogels	LUT	HHL hydrogel, a self-assembled HA/His/LUT structure, provides pH responsiveness and CD44 targeting. Oral delivery enhances GI stability, prolongs retention, suppresses TNF-α/IL-1β/IL-6, upregulates ZO-1/Occludin/Claudin-1, modulates microbiota for SCFA production, and repairs barrier/immune dysfunction in UC.	[250]
Natural carrier-free SAMs	Quercetin	Nanoribbons	Quercetin	Quercetin SNRs, self-assembled nanoribbons, exhibit antioxidant activity. Oral delivery enables GI stability, targets inflamed epithelium via electrostatic interactions, suppresses TNF-α/IL-6, repairs intestinal barrier, and modulates immunity without altering microbiota, demonstrating efficacy in IBD models.	[251]
Natural carrier-free SAMs	Berberine (BBR), Hesperetin (HST)	Spherical NPs	BBR, HST	BBR-HST NPs, self-assembled spheres (∼176 nm), orally target colon with GI resistance. They suppress TNF-α/IL-6, enhance tight junction proteins (e.g., Claudin-1/ZO-1), regulate microbiota, and ameliorate UC by overcoming GI barrier, epithelial dysfunction, and immune/microbial dysregulation.	[252]

Colon-specific delivery systems based on SAMs can be designed to achieve targeted drug release in the colon, either via pH-responsive polymers that stay stable in the acidic stomach but dissolve in the neutral-to-alkaline milieu of the lower intestine and colon or via coatings degradable by enzymes secreted by colonic microbiota [244]. Additionally, mucoadhesive components such as saccharide-based chitosan can prolong the residence time of drug carriers at inflamed mucosal sites, thereby boosting drug absorption and efficacy [245]. For IBD therapy, saccharide-based SAMs hold particular promise because many polysaccharides possess inherent anti-inflammatory activities and can function as prebiotics to selectively foster the proliferation of beneficial gut bacteria, which helps restore microbial homeostasis and enhance the production of short-chain fatty acids like butyrate—a key energy source for colonocytes that plays a vital role in preserving intestinal barrier integrity [246]. SAMs can also be engineered to deliver probiotics while shielding them from gastric acid to ensure their viability in the colon. Moreover, encapsulating potent anti-inflammatory agents (e.g., corticosteroids, cyclosporin A) in SAMs enables the attainment of high local drug concentrations at inflammatory sites, which minimizes systemic absorption and related adverse effects. Even some SAMs can passively target inflamed tissues by virtue of their leaky structure or can be actively directed to inflamed cells [244].

### 6.4 Psoriasis therapy

Skin diseases like eczema, psoriasis, and acne are common health problems. Psoriasis is a chronic, immune-mediated inflammatory skin disease characterized by red, scaly plaques, Which causes physical suffering and may trigger psychological disorders while bringing economic burdens from continuous treatment. Current therapies focus on anti-inflammation, immune response modulation, skin barrier repair, and inhibition of abnormal keratinocyte proliferation. Treatment is challenging due to the formidable barrier properties of the skin and the complex interplay between the immune system and skin cells. Topical therapy, the first-line treatment for psoriasis, must overcome significant physical and immunological barriers to be effective. The primary physical barrier is the stratum corneum (SC), the epidermis’ outermost layer. In healthy skin, the SC effectively restricts substance penetration, but psoriasis exhibits paradoxical SC dysfunction: though pathologically compromised and permeable to water, keratinocyte hyperproliferation causes marked hyperkeratosis, resulting in a thickened, scaly SC that acts as a major barrier to topical drug penetration [253]. Additionally, psoriatic plaques are heavily infiltrated by activated immune cells such as T cells, dendritic cells, and macrophages. These cells secrete various pro-inflammatory cytokines (e.g., TNF-α, IL-23, IL-17) that drive disease progression, and effective therapies must either suppress the localized immune response or deliver drugs directly to these pathogenic cells [254]. Moreover, these cytokines stimulate keratinocyte hyperproliferation, inducing the characteristic epidermal thickening and plaque formation, rendering the inhibition of this hyperproliferation a key therapeutic objective [255]. SAMs provide novel solutions for topical drug delivery in psoriasis by enhancing skin penetration, targeting pathogenic cells, and modulating the inflammatory microenvironment. Table 6 summarizes the applications of SAMs as DDSs in tackling key obstacles to psoriasis therapy.

**Table 6:** SAMs for psoriasis treatment.

SAMs	Building Blocks	Morphology	Drugs	Therapeutic Mechanisms	Ref.
DES	Hyaluronic acid-lithocholic acid (HA-LCA)	Spherical NPs	HA, LCA	HA-NPs, self-assembled spherical NPs, enable transdermal delivery to psoriatic lesions. They target macrophages via TLR4, suppress M1 polarization and pro-inflammatory cytokines, enhance barrier proteins, and restore skin function.	[21]
DES/Polysaccharide SAMs	HP-β-CD, Levulinic acid (Lev), Chitosan (CS)	Gel	Resveratrol (RES)	DES/CS, a supramolecular adhesive soft film to break skin barrier, transdermal delivery RES by enhancing skin penetration/retention. It exhibits anti-inflammatory effects via suppressing IL-23/IL-17 axis.	[108]
Amphiphilic block copolymer materials	Berberine (BBR), Ferulic acid (FA)	Spherical NPs	BBR, FA	BBR and FA self-assemble to BFN NPs, which enable transdermal delivery, provide antioxidant/anti-inflammatory effects, modulate oxidative stress/microbiota, and ameliorate psoriasis.	[257]
DNA origami	Four ssDNA	Tetrahedral NPs	miRNA-125b mimic	FNA-miR-125b framework nucleic acid nanostructure enhances miRNA stability and transdermal delivery, inhibits keratinocyte proliferation, and improves immune barrier in psoriasis.	[258]
LNPs	ALC-0315/DOTAP, DSPC, Chol, DMG-PEG2000	Spherical LNPs	Budesonide, STAT3 siRNA	C8B2 LNPs, a spherical NPs with budesonide, enables topical si-Stat3 delivery. It penetrates the skin barrier, suppresses STAT3 signaling, reduces TNF-α/IL-6, and ameliorates immune dysregulation in psoriasis models.	[259]
Amphiphilic block copolymer materials	Glycyrrhizic acid (GA), Egg yolk lecithin, Chlorin e6 (Ce6)	Core-shell NPs	Ce6, GA	GC NPs/QCS-C nanosystem with core-shell structure adheres to skin, co-delivers Ce6/GA, enables photodynamic/anti-inflammatory therapy for psoriasis.	[260]
Amphiphilic block copolymer materials	Bilirubin, V9302	Spherical NPs	Bilirubin, V9302	BVn NPs self-assemble from bilirubin/V9302, regulate glutamine metabolism/oxidative stress, and inhibit keratinocyte proliferation via transdermal delivery.	[261]
Amphiphilic block copolymer materials	Oxymatrine, Lauric acid	Reverse micelles	Triamcinolone acetonide	DES-RM reverse micelles self-assemble to enhance drug solubility and transdermal delivery, overcoming skin barrier for psoriasis-related immune dysregulation therapy.	[262]
ILs	Choline and geranic acid (CAGE), Choline and phenylpropanoic acid (CAPA)	Ionic aggregate	NFKBIZ siRNA	IL-siRNA nanocomplex uses ionic liquids to stabilize siRNA, enhances transdermal delivery for NFKBIZ gene silencing, and modulates immune barrier in psoriasis.	[263]
DNA origami	Four ssDNA	Tetrahedral NPs	Tannic acid (TA), siRNA	STT nanocomplex enables smart siRNA delivery via cascade-responsive design, transdermally silences NF-κB p65 to ameliorate immune dysregulation in psoriasis.	[264]

Amphiphilic SAMs such as nanomicelles act as skin penetration enhancers: their lipid-like components fluidize the intercellular lipid matrix of the SC to create pathways for drug permeation, while nanoparticle formulations improve transdermal drug retention to achieve sustained therapeutic effects and minimize systemic absorption. Furthermore, combining such SAMs with physical approaches like microneedle-mediated transient micropore formation in the SC can drastically boost drug delivery efficiency [256]. Moreover, SAMs can be engineered for targeted delivery to the pathogenic immune cells driving psoriasis, for instance, HA-based nanoparticles can selectively bind to receptors such as CD44 and TLR4 that are highly expressed on activated macrophages in psoriatic skin, thus enabling targeted delivery of anti-inflammatory agents to the key mediators of inflammation [21]. Additionally, SAMs can be constructed from materials with intrinsic therapeutic properties, including natural product-derived self-assembled nanoparticles that exert concurrent antioxidant, antimicrobial, and anti-inflammatory effects [257], as well as DNA-based SAMs that deliver therapeutic oligonucleotides such as miRNA to modulate the gene expression profiles of skin and immune cells, thereby quelling inflammation and normalizing keratinocyte proliferation [258].

### 6.5 Wound infection therapy

Wound infection is a common traumatic complication (e.g., bacterial, fungal, and mixed infections), which leads to local redness, swelling, suppuration, and even systemic infection or organ failure. Treatment focuses on antibacterial sterilization, controlling inflammation, clearing necrotic tissue, and promoting healing. The rise of antibiotic-resistant bacteria, such as methicillin-resistant *Staphylococcus aureus* (MRSA), has created an urgent need for new therapeutic strategies that can effectively eliminate pathogens and promote tissue regeneration. The successful treatment of infected wounds requires overcoming microbial defenses while promoting a pro-healing microenvironment in host tissues. Pathogenic bacteria such as MRSA form wound-bed biofilms—bacterial communities encased in a self-secreted extracellular polymeric matrix that acts as a strong physical barrier, blocking antibiotics and shielding bacteria from host immune cells, making biofilm eradication a key challenge in chronic wound care [265]. Infected wounds also exhibit sustained excessive inflammation, where high levels of cytokines, proteases and ROS generate a toxic microenvironment that damages host cells, degrades the extracellular matrix (ECM) and impairs key healing cells (fibroblasts, keratinocytes), perpetuating a non-healing state [266]. Furthermore, persistent infection and chronic inflammation disrupt coordinated wound healing, trapping wounds in the inflammatory phase and blocking progression to the proliferative and remodeling phases needed for new tissue formation [267]. Supramolecular SAMs provide strategies for wound infection therapy. Table 7 summarizes the applications of SAMs as DDSs in tackling key obstacles to wound infection therapy.

**Table 7:** SAMs for wound infection treatment.

SAMs	Building Blocks	Morphology	Drugs	Therapeutic Mechanisms	Ref.
Natural carrier-free SAMs	Berberine (BBR), Chlorogenic acid (CGA)	Spherical NPs	BBR, CGA	BBR and CGA self-assemble into NPs, which inhibit MRSA through disrupting bacterial membranes, inhibiting biofilms, and down-regulating drug resistance genes.	[59]
Natural carrier-free SAMs	Gallic acid (GA)	Fibrillar hydrogels	GA	GA self-assemble into fibrous hydrogels. Providing antibacterial, anti-inflammatory, and promoting healing, effectively accelerating wound healing.	[82]
Natural carrier-free SAMs	Glycyrrhizic acid (GA), Puerarin (PUE)	Nanofiber hydrogel	GA, PUE	GA and PUE co-assembly into injectable and self-healing hydrogel. Its nanofiber structure exhibits selective antibacterial activity against staphylococcus aureus.	[80]
MPN	EGCG, Au^3+^	Spherical NPs	EGCG-Au	EGCG-Au NPs effectively penetrate and eliminate drug-resistant bacterial biofilms through mechanisms such as photothermal, ROS and quinone proteins. Promoting anti-inflammatory and healing.	[273]
LNPs	Various lipids	Spherical LNPs	Lipopeptide R6F	R6F, a fluorinated peptide, effectively kill bacteria by disrupting the bacterial membrane, inhibiting the respiratory chain and cell wall synthesis.	[268]
Peptide SAMs	Nap-FFKKK Peptide	Nanofiber hydrogel	Nap-FFKKK, CUR	The pH-responsive peptide assembled into hydrogel with nanofiber network, enhancing the stability of drug loading, it decomposes and releases drugs controllably in the acidic environment of infection, and damages bacterial membranes and biofilms.	[269]
MPN	Tannic acid (TA), Zn^2+^	Spherical NPs	CUR, TA/Zn	A polysaccharide self-healing hydrogel enhanced by TA-Zn nanospheres was constructed. it simultaneously achieves antibacterial, antioxidant, anti-inflammatory functions and promotes angiogenesis.	[271]
Polysaccharide SAMs	Chitosan (CS), Puerarin (PUE)	Nanofiber hydrogel	CS@PUE	CS@PUE hydrogel with nanofiber provides antibacterial and immunomodulatory, and promotes the healing of infected wounds.	[272]
Peptide SAMs	Silk fibroin	Nanofibrillar microgels	Ag-NPs	Nanofiber microgels, loaded with silver NPs, achieves a two-step mechanism of bacterial enrichment and local efficient killing, while reducing the toxicity of silver.	[274]
Polysaccharide SAMs	Mono-carboxyl corrole (MCC), chitosan (CS)	Core-shell NPs	MCC, CS	MCC/CS NPs, with core-shell structure, target bacteria and promote angiogenesis. exhibiting highly efficient photothermal sterilization effects.	[275]
Polysaccharide SAMs	Stearic acid-bletilla striata polysaccharide (SA-BSP)	Nanomicelles	Azithromycin, BSP	SA-BSP self-assembled nanomicelles synergistically treat acute and chronic infected wounds through antibacterial effects, regulation of macrophage M1/M2 polarization, promotion of angiogenesis and collagen deposition.	[276]
DNA origami	Single-stranded DNA with Y-scaffolds and L-linkers	Hydrogel	(LKKL)_3_ Peptide	DNA hydrogel loads (LKKL)_3_ peptides through electrostatic adsorption and responds to the release of bacterial nucleases, achieving antibacterial, anti-inflammatory and promoting healing.	[277]
DNA origami	DNA duplex (M13)	Rectangular nanosheet	DNAzyme(G4/hemin) Levofloxacin	DNA origami is equipped with targeting aptamers and DNA enzymes. destroying bacterial membrane and releasing antibiotics, achieving precise sterilization and promoting healing.	[278]

SAMs w fabricated with potent antibacterial activity to eradicate bacteria and biofilms. Cationic peptide-functionalized SAMs electrostatically target the anionic bacterial cell membrane, penetrate and disrupt membrane integrity, thereby inducing bacterial cell lysis [268,269]. This physical mode of action confers a lower propensity for resistance development compared to conventional antibiotics. Stimuli-responsive hydrogel-integrated SAMs serve as controlled antimicrobial delivery systems: pH-responsive formulations release their payloads in the slightly acidic microenvironment of infected wounds, while enzyme-responsive systems trigger drug release upon exposure to bacterial enzymes (e.g., DNase) that are abundant in biofilms [269,270]. Metal-coordinated SAMs act as reservoirs for antimicrobial metal ions (e.g., Zn^2+^, Ag^+^), mediating sustained antibacterial effects [271]. Beyond direct bactericidal activity, SAMs modulate the wound microenvironment to create a pro-healing niche. Hydrogel-based SAMs maintain a moist wound bed, absorb excess exudate, and form a physical barrier against secondary contamination. Furthermore, natural polymers utilized in SAMs, including chitosan and hyaluronic acid, exhibit excellent biocompatibility and biodegradability. These polymers actively promote tissue regeneration by enhancing cell proliferation, migration, and angiogenesis, thereby accelerating wound re-epithelialization [272].

## 7 Conclusion and outlook

### 7.1 Conclusion

After over seven decades of development, modern drug delivery technology has created numerous systems to enhance therapeutic efficacy. However, few systems have received FDA approval or clinical application. Since the concept of supramolecular self-assembly was proposed, research on DDS has progressed rapidly. This article traces the development trajectory of SAMs and identifies related SAMs that have been approved for marketing and clinical application. Notably, most emerging SAMs lack clinical translation due to insufficient industry standards, with research remaining at laboratory level. Therefore, this article systematically reviews research advances in supramolecular SAMs, emphasizing their potential to drive innovation in the DDS field. We summarize assembly mechanisms and core functional advantages to deepen understanding of these materials. Simultaneously, the article explores innovative design strategies for SAMs as DDSs and their potential in disease treatment, providing references for rational design of self-assembling delivery systems.

DDS constructed from SAMs have controllable size distributions and tunable structures, with programmed design at their core. Carrier-type SAMs achieve multifunctional integration through engineered modification, demonstrating unique capabilities in DDS—integrating various functions and enhancing material properties. Small-molecule self-assembly strategies focus on designing therapeutic or functional molecules while possessing self-assembly capability for carrier-free delivery. Furthermore, by co-loading drugs or functional nanomaterials within self-assembled nanostructures, combined applications expand SAMs’ scope in DDSs.

Among optimization design strategies, programmed design is particularly crucial. DNA and peptide molecular sequence design shows significant advantages, while programmed design of other molecules (such as introducing pH, ROS, enzyme responsiveness, or functional/therapeutic properties through modification) remains indispensable. Recent research has widely applied these optimization strategies. Future optimization will focus on intelligent responsive regulation to overcome single stimuli limitations for precise drug delivery control. (ⅰ) Intelligent Mult-Stimuli-Responsive Systems: Integrate environmental stimuli (weakly acidic pH, high ROS concentration, specific enzyme overexpression in tumor tissues) to construct drug release systems with spatiotemporal specificity. Through molecular structure design, SAMs can respond to these signals at tumor sites, precisely controlling the sequence, location, and timing of drug release. (ⅱ) Intelligent *in Situ* Self-Assembly: Design molecules that self-assemble at specific sites, using molecular recognition events (such as antibody-antigen, receptor-ligand binding) in target tissues to induce molecular structural transformations and form stable nanoassemblies in situ. This prevents premature disassembly during circulation, improving targeting and bioavailability. (ⅲ) Externally Driven Controllable Self-Assembly: Use external stimuli like temperature, light, magnetic fields, and ultrasound to control the self-assembly process, enhancing stability and behavioral control for precision therapy [[279], [280], [281], [282]].

Combination therapy uses multiple drugs synergistically to reduce toxicity or enhance efficacy, aligned with traditional Chinese medicine. While DDS enables co-delivery of multiple drugs, traditional carriers have limited drug-loading capacity. Carrier-free delivery strategies increase drug-loading capacity, showing a new trend. Layer-by-layer self-assembly technology enables co-assembly of multiple components, constructing complex structures with multiple functions by integrating different self-assembly systems, achieving synergistic therapeutic effects [283,284]. For example, we envision a layer-by-layer system combining small molecules, metal ions, and DNA. Metal coordination provides stable support for the inner (core) small-molecule assembly and the externally coated DNA layer, forming a triple-layer core–shell structure. This composite assembly integrates photothermal/photodynamic therapy (PTT/PDT), chemotherapy (CT), and gene therapy, building a multifunctional synergistic therapeutic system. At the tumor site, the microenvironment is disrupted via the photothermal effect while releasing chemotherapeutic drugs and gene therapeutics, synergistically inhibiting tumor growth. With targeting modifications, these systems achieve precision therapy balancing safety and efficacy.

### 7.2 Outlook

Despite innovations in drug delivery technology, the industrialization of new-generation SAMs remains slow. Approved self-assembling DDSs mainly include liposomes (Doxil®), polymeric micelles (Genexol®-PM), cyclodextrin complexes (Sporanox®), drug co-crystals (Entresto®), albumin nanoparticles (Abraxane®), and VLPs (Recombivax HB®). However, emerging complex SAMs such as DNA nanomaterials, peptide-based nanomaterials, and MOFs remain largely confined to laboratories. The core value of supramolecular chemistry lies in its dynamic and reversible self-assembly properties, which endow materials with unique stimuli-responsiveness and environmental adaptability. Yet this dynamism creates translational bottlenecks in the complex, harsh physiological environment. Key challenges include: (ⅰ) instability from disassembly under shear forces and pH fluctuations; (ⅱ) immunogenicity induced by exogenous nanomaterials; (ⅲ) unpredictable biodistribution and inadequate tumor targeting precision; and (ⅳ) difficulties standardizing and scaling up manufacturing for industrial application.

The future development of supramolecular chemistry should center on an engineering-driven paradigm. Specifically, stability and biocompatibility can be enhanced via three key strategies: (ⅰ) Post-assembly covalent locking: Introducing cysteine residues into peptides and forming intermolecular disulfide bonds through mild oxidation strengthens cross-linking and degradation resistance [285]. (ⅱ) Strengthening core non-covalent interactions (hydrophobic/π-π): Molecular modeling screens for strong hydrophobic groups (e.g., tryptophan, fluorinated chains) or π-conjugated units to enhance these interactions [285]. (ⅲ) Next-generation stealth polymer modifications (replacing PEG with poly-sarcosine) to form a hydrophilic corona and reduce immunogenicity. In addition, overcoming targeted delivery barriers relies on spatiotemporal precision control: (ⅰ) Multi-ligand synergistic targeting: co-modifying carriers with folic acid, HER2 aptamers, etc., to improve tumor specificity [286]. (ⅱ) Tumor microenvironment (TME)-triggered in situ assembly: Designing precursors with substrates for TME-overexpressed enzymes (e.g., MMPs) that assemble locally into nanofibers after enzymatic cleavage [287,288]. Finally, establishing stable, controllable large-scale production requires deep integration of the *Quality by Design* framework with continuous manufacturing technologies. For example, Design of Experiments maps process parameters (e.g., mixing speed, solvent ratio) against particle size to define a predictable, controllable design space [289,290]. In essence, the engineering-driven paradigm emphasizes proactive design and optimization of supramolecular systems to overcome inherent limitations and pave the way for practical applications.

Deep cross-disciplinary integration drives the advancement of SAMs in DDS. Integrating theories and technologies from different disciplines helps overcome challenges, accelerating the research and development, and application processes. Machine learning algorithms analyzing molecular structure, assembly condition, and performance data can rapidly screen promising self-assembling molecules and predict their behavior and performance, significantly enhancing design efficiency and precision [291]. Furthermore, combining theoretical computational models, such as molecular dynamics simulations and quantum chemical calculations, offers insights into mechanisms of interaction, formation pathways, and structural evolution at the atomic level, providing a basis for rational design. Self-assembling nanomaterials binding with biological targets can form electrochemical sensing platforms, where structural changes translate biomolecular reactions into electrical signals for diagnosis [292,293]. The key to future development lies in integrating theory and engineering practice. There is an urgent need to establish standardized production processes for SAMs, characterization systems for intermolecular forces, and quality control standards for stable assemblies. These measures will promote a cycle from basic research to technological translation to clinical application, promoting industrialization and contributing to human health.

## CRediT authorship contribution statement

**Hongwei Fu:** Writing – original draft, Validation, Methodology, Investigation, Formal analysis, Data curation, Conceptualization. **Weihao Gao:** Writing – original draft, Validation, Methodology, Investigation, Data curation. **Yixuan Tang:** Writing – original draft, Validation, Methodology, Investigation, Data curation. **Xiangli Liu:** Validation, Supervision, Methodology. **Mi Wang:** Validation, Methodology, Investigation. **Jichuan Zhang:** Validation, Investigation, Formal analysis, Data curation. **Tianqi Liu:** Writing – review & editing, Visualization, Resources, Project administration. **Jiaheng Zhang:** Writing – review & editing, Supervision, Project administration, Funding acquisition, Conceptualization.

## Data availability statement

There is no new data generated or analyzed, and the data supporting this work can be found in the referenced sources.

## Ethics approval and consent to participate

This article does not involve ethical review.

## Declaration of competing interest

The authors declare that they have no known competing financial interests or personal relationships that could have appeared to influence the work reported in this paper.
